# Supplementary material for: Prophage induction can facilitate the in vitro dispersal of multicellular Streptomyces structures
Source: PLoS Biol. 2024 Jul 25;22(7):e3002725. doi: 10.1371/journal.pbio.3002725 (PMC11302927; doi:10.1371/journal.pbio.3002725)
Supplement: S1 Fig — (PDF) [file pbio.3002725.s001.pdf]

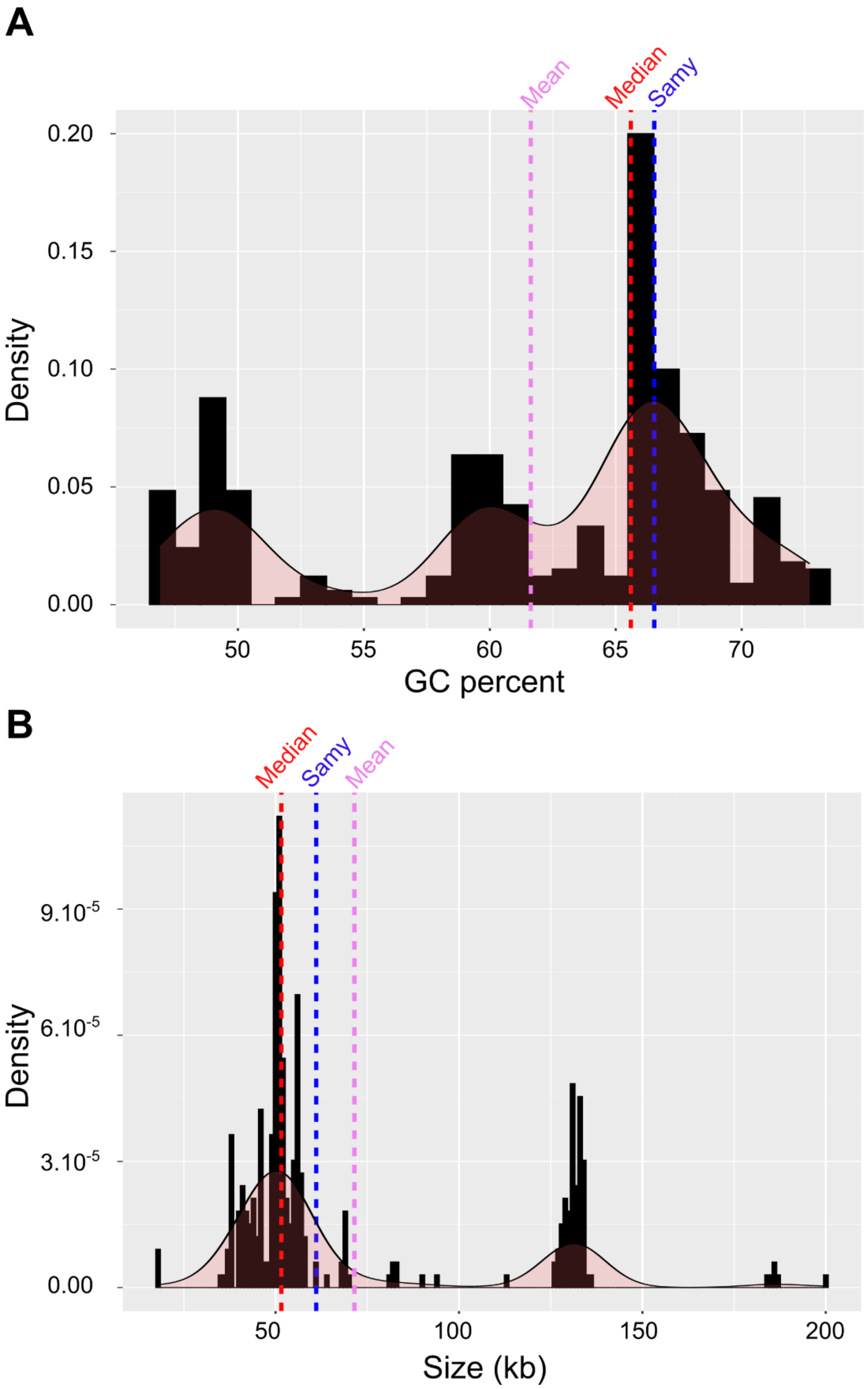

S1 Figure: GC content (A) and size (B) of a panel of 330 *Streptomyces* phages

This analysis was conducted on a panel of 330 sequenced phages, including Samy, listed in the Actinophage Database, NCBI and/or ICTV databases (**S1 Table**). Since the distribution of values is multimodal, information about both the mean (pink dashed line) and median (red dashed line) was taken into account to compare to Samy (blue dashed line). The density plots were generated using a bin width of 1 and 1000 in panels A and B, respectively. The density curves are indicated in each panel. The data and scripts underlying these panels can be found in **S1 Table** and **S2 Data**, respectively.
