# Supplementary material for: Prophage induction can facilitate the in vitro dispersal of multicellular Streptomyces structures
Source: PLoS Biol. 2024 Jul 25;22(7):e3002725. doi: 10.1371/journal.pbio.3002725 (PMC11302927; doi:10.1371/journal.pbio.3002725)
Supplement: S2 Fig — (PDF) [file pbio.3002725.s002.pdf]

A

Condition name	Medium type	Medium composition	Time point (h)	GEO accession number
MP24	Liquid	MP5	24	GSE162865
MP30	Liquid	MP5	30	GSE162865
MP36	Liquid	MP5	36	GSE162865
MP48	Liquid	MP5	48	GSE162865
MP72	Liquid	MP5	72	GSE162865
Y24	Liquid	YEME 10% saccharose	24	GSE162865
Ycongo24	Liquid	YEME 10% saccharose + 1µg/ml congocidine	24	GSE162865
Y48	Liquid	YEME 10% saccharose	48	GSE162865
MM	Solid	Minimal Medium 0.5 % Mannitol	30	GSE232795
NAG	Solid	MM + 25 mM N-Acetyl-Glucosamine	30	GSE232795
SAF	Solid	SAF	30	GSE232795
ONA	Solid	Oxoid Nutrient Agar	30	GSE232795
HT	Solid	Hickey-Tresner agar	30	GSE232795

B

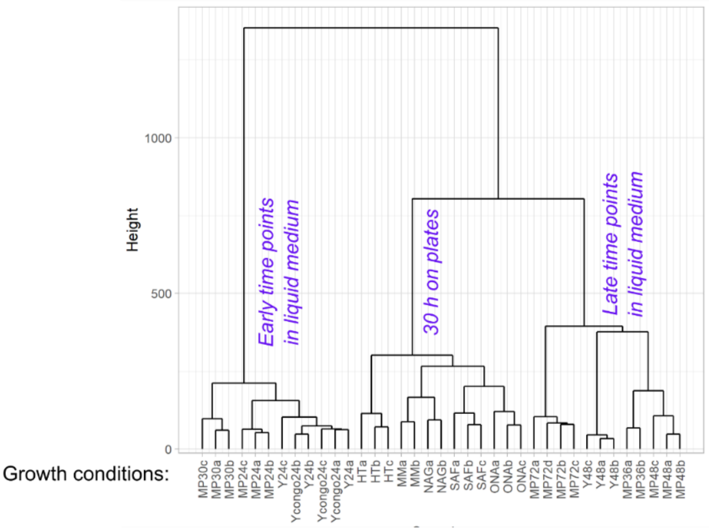

C

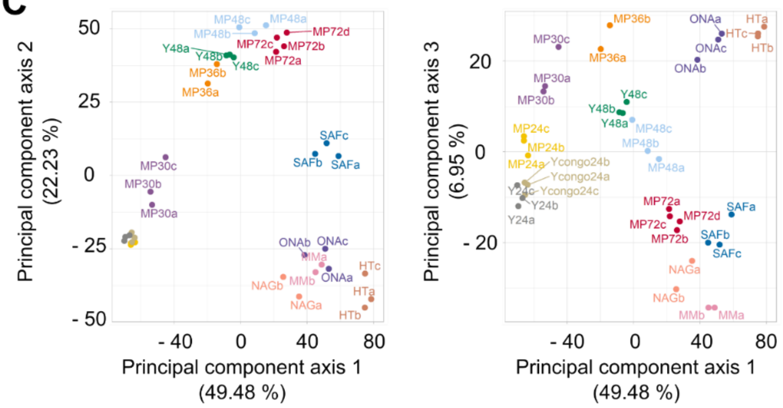

S2 Figure: Multidimensional analyses of the transcriptomes of *S. ambofaciens* ATCC 23877 grown under various conditions

A. **Main characteristics of the various growth conditions analyzed in this study**.
B. **Ascending hierarchical classification** (Euclidean distance, Ward criterion) of the whole data set (37 samples). Transcriptomes form clusters based on biological conditions specified in blue near each node. The acronyms and colors indicate the condition (see panel A) with a distinct letter (a, b, c or d) for each replicate.
C. **Principal component analysis** (PCA) of the whole data set, with percentages of variance associated with each axis. The acronyms and colors indicate the condition (see panel A) with a distinct letter (a, b, c or d) for each replicate.

The data and scripts underlying these panels can be found in **S1 Data** and **S2 Data**, respectively. The analyses were performed on raw counts using the SARTools DESeq2-based R pipeline (1). The statistical report generated by this pipeline is presented in **S3 Data**.

Reference:

1. Varet H, Brillet-Gueguen L, Coppee JY, Dillies MA. SARTools: A DESeq2- and EdgeR-Based R Pipeline for Comprehensive Differential Analysis of RNA-Seq Data. PLoS One. 2016;11(6):e0157022.
