# Supplementary material for: Prophage induction can facilitate the in vitro dispersal of multicellular Streptomyces structures
Source: PLoS Biol. 2024 Jul 25;22(7):e3002725. doi: 10.1371/journal.pbio.3002725 (PMC11302927; doi:10.1371/journal.pbio.3002725)
Supplement: S3 Fig — (PDF) [file pbio.3002725.s003.pdf]

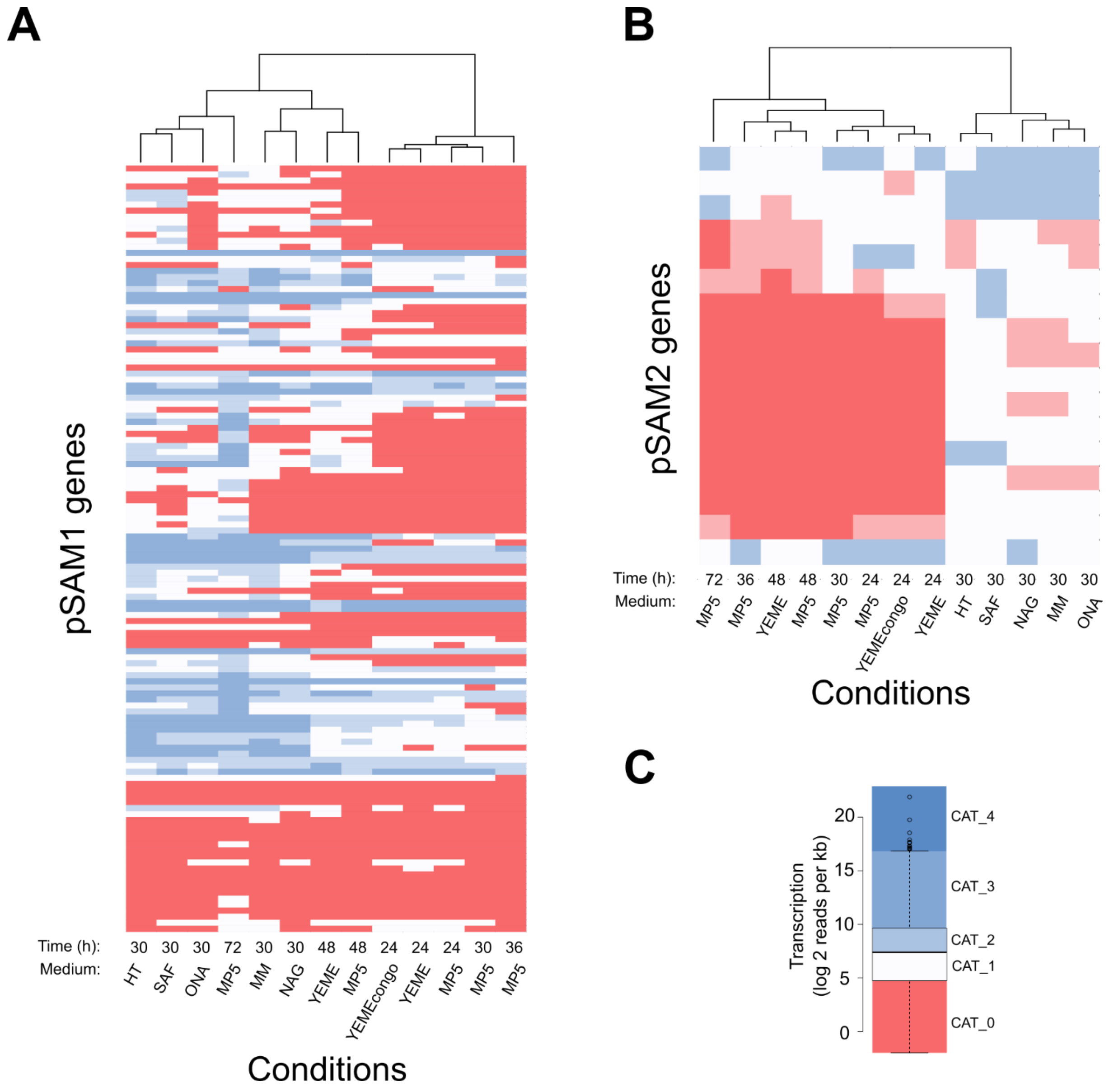

S3 Figure: Heatmaps of pSAM1 (A) and pSAM2 (B) transcriptomes in different growth conditions

RNA-seq data (DESeq2 normalized number of reads per kb per gene, log2) were categorized in a color scale reflecting their expression relative to whole genome expression in each condition, as illustrated in panel C. Each line represents a gene, ranked according to order on the genome. Growth conditions (columns) are ranked by hierarchical clustering according to pSAM1 and pSAM2 transcriptomes, in panels A and B, respectively. The data underlying these panels can be found in **S1 Data**.
