# Supplementary material for: Prophage induction can facilitate the in vitro dispersal of multicellular Streptomyces structures
Source: PLoS Biol. 2024 Jul 25;22(7):e3002725. doi: 10.1371/journal.pbio.3002725 (PMC11302927; doi:10.1371/journal.pbio.3002725)
Supplement: S4 Fig — (PDF) [file pbio.3002725.s004.pdf]

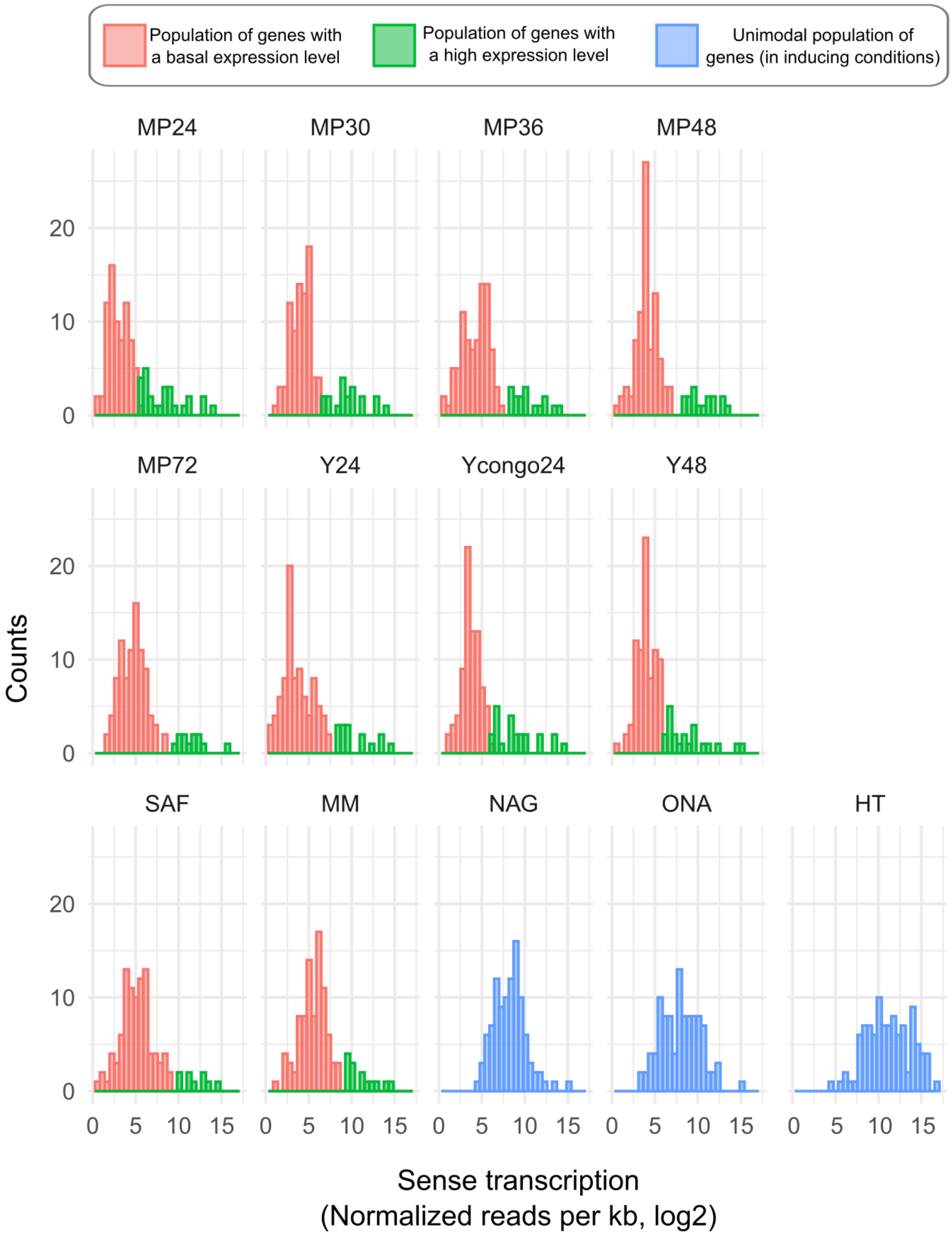

S4 Figure: Samy expression profile under the different conditions studied

The expression level distribution of Samy genes (n = 102) is presented in each condition (described in **S2 Fig** and **S6 Table**). A dedicated R package ('mClust') was used to identify the bimodal distributions and classify genes accordingly. In non-inducing conditions (*i.e.* all conditions except HT, ONA, NAG), a bimodal profile was observed, genes being categorized in the basal (red) or high expression level (green) population. In 'HT', 'ONA' and 'NAG' conditions, the distribution of Samy gene expression was unimodal (population in blue). The data and scripts underlying these panels can be found in **S1 Data** and **S2 Data**, respectively.
