# Supplementary material for: Prophage induction can facilitate the in vitro dispersal of multicellular Streptomyces structures
Source: PLoS Biol. 2024 Jul 25;22(7):e3002725. doi: 10.1371/journal.pbio.3002725 (PMC11302927; doi:10.1371/journal.pbio.3002725)
Supplement: S5 Fig — (PDF) [file pbio.3002725.s005.pdf]

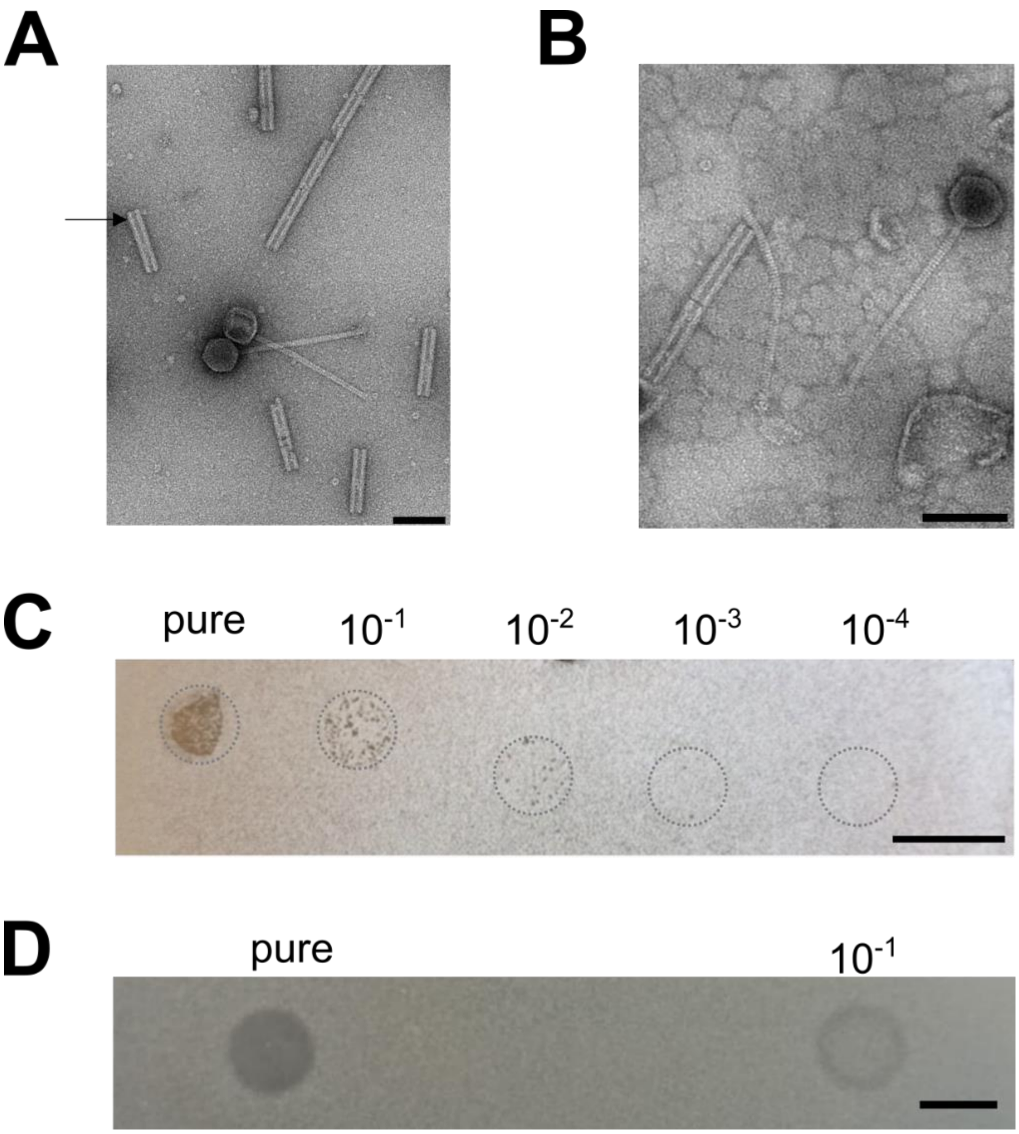

S5 Figure: Samy phage morphology and infection assays

A. Imaging of Samy phage produced in BM medium by transmission electron microscopy. *S. ambofaciens* ATCC 23877 was grown during 4 days in BM medium. The supernatant was concentrated by CsCl-gradient ultracentrifugation. Viral particles were negatively stained with uranyl acetate. Two Samy phages surrounded by regular double tubular structures (arrow) appearing to originate from a larger structure, composed of subunits of these. These structure have been described as phage tail-like nanostructures and identified as extracellular contractile injection systems (1). Scale bar: 100 nm.

B. Imaging of Samy phage produced in HT medium by transmission electron microscopy. *S. ambofaciens* ATCC 23877 was grown during 3 days in HT medium. Supernatant was harvested and concentrated by an iodixanol gradient. The sample was negatively stained with uranyl acetate. Samy virions and extracellular contractile injection systems were also observed. Scale bar: 100 nm.

C. Infection of *S. lividans* TK24 by Samy phage. Five µl of serial dilutions of Samy phage produced after 4 days-growth in BM medium were concentrated by centrifugation and spotted on a lawn of *S. lividans* TK24 spores poured onto an SFM plate. The picture was taken after one week of growth at 30°C. Spotted sites are framed by a grey circle. Viral plaques appear as small holes in the *S. lividans* lawn (white). Scale bar: 1 cm.

D. Infection of *S. ambofaciens* ATCC 23877 by Samy phage. Five µl of serial dilutions of Samy phage produced after 4 days-growth in BM medium were concentrated by centrifugation and applied onto MNB agar plates supplemented with 0.5% glucose, 10 mM $MgCl_2$, and 8 mM $CaCl_2$ prior to pouring MNB soft agar containing spores. The picture was taken after one day of growth at 30°C. Scale bar: 0.5 cm.

Reference:

1. Nagakubo T, Yamamoto T, Asamizu S, Toyofuku M, Nomura N, Onaka H. Phage tail-like nanostructures affect microbial interactions between *Streptomyces* and fungi. Sci Rep. 11 oct 2021;11(1):20116.
