# Supplementary material for: Prophage induction can facilitate the in vitro dispersal of multicellular Streptomyces structures
Source: PLoS Biol. 2024 Jul 25;22(7):e3002725. doi: 10.1371/journal.pbio.3002725 (PMC11302927; doi:10.1371/journal.pbio.3002725)
Supplement: S6 Fig — (PDF) [file pbio.3002725.s006.pdf]

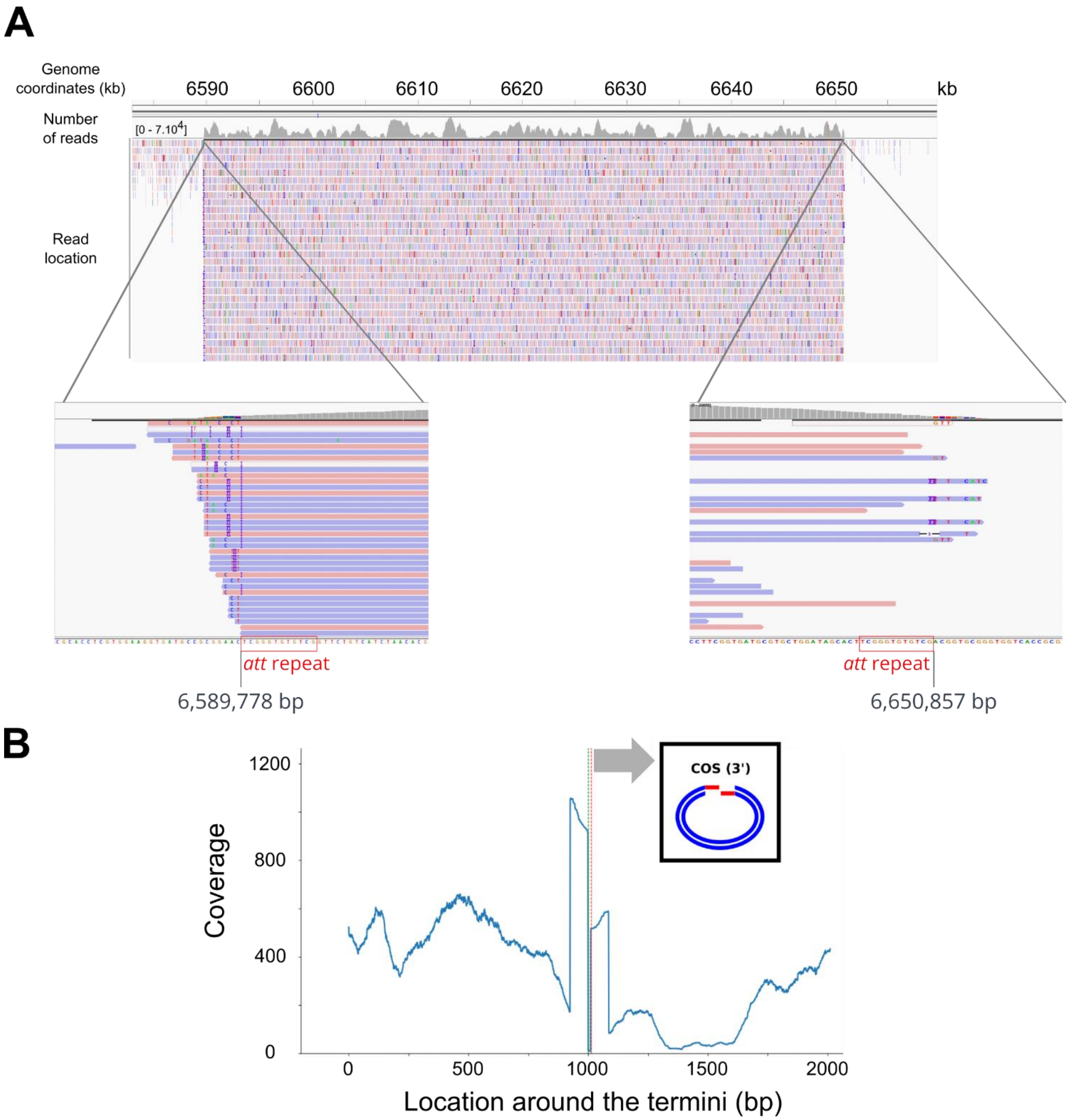

S6 Figure: Results of high-throughput sequencing of the double-stranded DNA virome of *Streptomyces ambofaciens* ATCC 23877 grown 4 days in BM medium

A. Sequence coverage around Samy prophage region. The coordinates of *S. ambofaciens* ATCC 23877 chromosome are indicated. The inserts present the focus on the regions surrounding the repeats contained in the *att*L and *att*R sites. The data were visualized using the Integrative Genome Viewer (v2.8.0) software.

B. Sequence coverage at termini positions identified by PhageTerm (1). Exact termini positions are represented by dotted lines (Red: left; Green: right). The predicted cohesive sequence (*cos*) is: CGTTAAGGTGC (from 6,593,965 to 6,593,975 bp position on *S. ambofaciens* ATCC 23877 chromosome). The analysis was obtained by PhageTerm (1) run from the Galaxy server (https://galaxy.pasteur.fr).

Reference:

1. Garneau JR, Depardieu F, Fortier LC, Bikard D, Monot M. PhageTerm: a tool for fast and accurate determination of phage termini and packaging mechanism using next-generation sequencing data. Sci Rep. 2017;7(1):8292.
