# Supplementary material for: Prophage induction can facilitate the in vitro dispersal of multicellular Streptomyces structures
Source: PLoS Biol. 2024 Jul 25;22(7):e3002725. doi: 10.1371/journal.pbio.3002725 (PMC11302927; doi:10.1371/journal.pbio.3002725)
Supplement: S7 Fig — (PDF) [file pbio.3002725.s007.pdf]

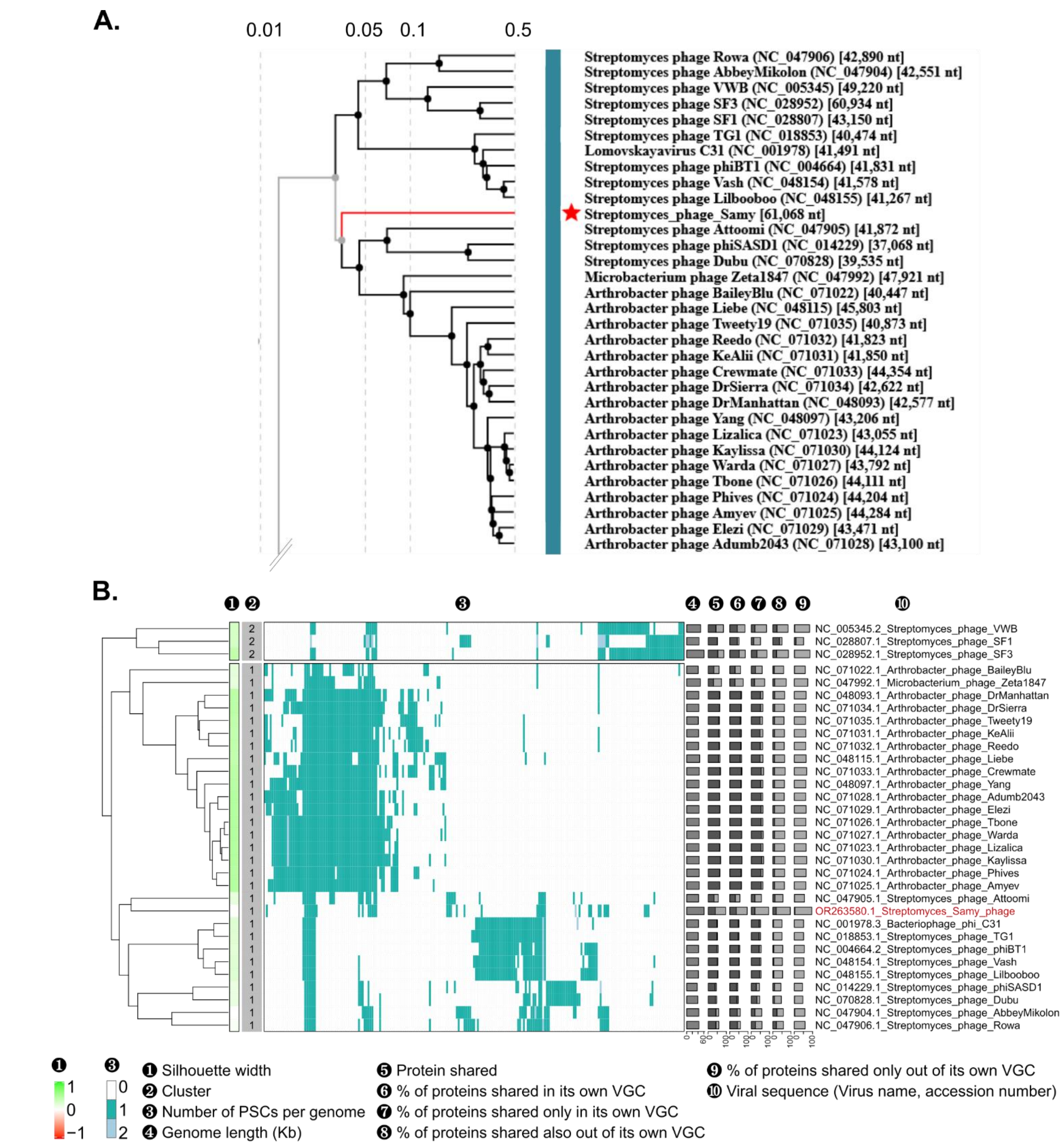

S7 Figure: Clustering of Samy with PhiC31 and other actinophages

A. **Local view of the proteomic tree of viruses grouping with Samy using VIPTree**. This analysis was conducted via the VIPTree (1) web server, comparing Samy (red star) against all reference viral genomes of the GenomeNet/Virus-Host DB (2). Here, only the relevant region of the tree with the clusters of phages closest to Samy are displayed. Branch lengths (similarity scores, S_G) are log-scaled and indicated above dashed grey lines.

B. **Viral clustering outputted by VirClust**. The genome clustering of all genomes presented in the panel A was performed via the VirClust web server (3) and based on protein super clusters (PSCs). The resulting tree was split into viral genome clusters (VGCs) using a 0.9 intergenomic distance threshold. The heat map represents the PSC distribution in the viral

genomes. The color scale indicates the number of copies per genome (from 0 to 2). The percentage of PSCs shared with other genomes are indicated in dark grey.

References:

1. Nishimura Y, Yoshida T, Kuronishi M, Uehara H, Ogata H, Goto S. ViPTree: the viral proteomic tree server. Bioinformatics. 2017;33(15):2379-80.

2. Mihara T, Nishimura Y, Shimizu Y, Nishiyama H, Yoshikawa G, Uehara H, et al. Linking Virus Genomes with Host Taxonomy. Viruses. 2016;8(3):66.

3. Moraru C. VirClust—A Tool for Hierarchical Clustering, Core Protein Detection and Annotation of (Prokaryotic) Viruses. Viruses. 2023;15(4):1007.
