# Supplementary material for: Prophage induction can facilitate the in vitro dispersal of multicellular Streptomyces structures
Source: PLoS Biol. 2024 Jul 25;22(7):e3002725. doi: 10.1371/journal.pbio.3002725 (PMC11302927; doi:10.1371/journal.pbio.3002725)
Supplement: S8 Fig — (PDF) [file pbio.3002725.s008.pdf]

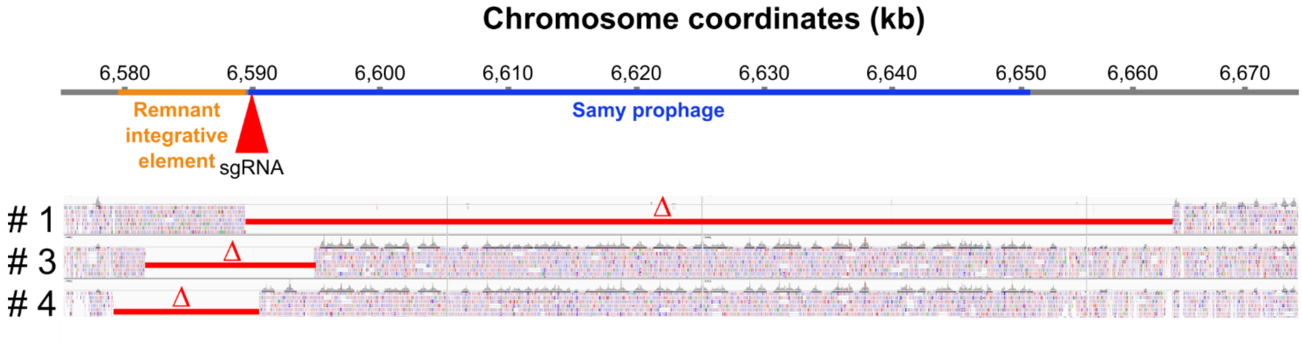

S8 Figure: Schematic representation of deletions generated by a CRIPSR-Cas9 approach targeting Samy prophage integrase

The Samy integrase gene position targeted the sgRNA used in this study is indicated by a red triangle. The location of the remnant integrative element and Samy prophage are colored in orange and blue, respectively. The position of the deletion (Δ) observed in each clone is highlighted by red lines. The visualization of the position of sequencing reads obtained for each clone was generated using the Integrative Genome Viewer (IGV). The precise position of each deletion is detailed in the **S4 Table**.
