# Supplementary material for: Prophage induction can facilitate the in vitro dispersal of multicellular Streptomyces structures
Source: PLoS Biol. 2024 Jul 25;22(7):e3002725. doi: 10.1371/journal.pbio.3002725 (PMC11302927; doi:10.1371/journal.pbio.3002725)
Supplement: S9 Fig — (PDF) [file pbio.3002725.s009.pdf]

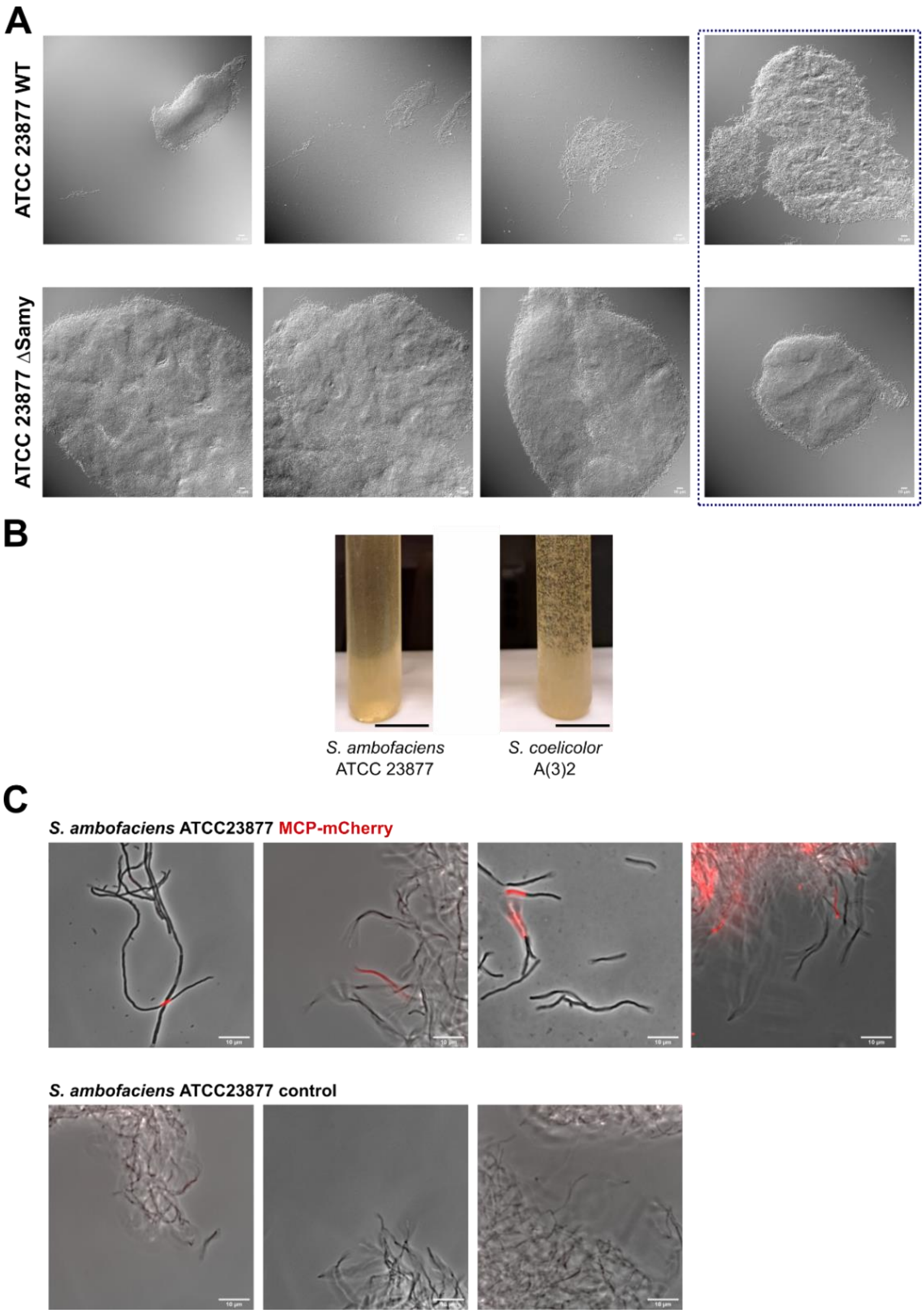

S9 Figure: Morphology of *S. ambofaciens* and *S. coelicolor* after 4 days growth in BM medium

A. **Microscopy of *Streptomyces* aggregates**. *S. ambofaciens* ATCC 23877 and its derivate CRISPR-deleted of Samy prophage (clone #3) were grown in BM medium and imaged using differential interference contrast. The framed images present rare observations (clusters in the WT strain, small pellets in the Samy-deleted strain). Additional fields of the experiment presented in **Fig 4.C** panel. Scale bar: 10 µm.

B. **Dispersed *versus* aggregated growth of *S. ambofaciens* ATCC 23877 and *S. coelicolor* A(3)2 strains in BM media.** The results are representative of the appearance of the most frequently observed cultures, as the quantity and size of cell clusters may vary from one experiment to the other. Scale bar: 1.5 cm.

C. **Pattern of Samy MCP-mCherry expression in *S. ambofaciens* colonies**. Bacteria encoding Samy MCP-mCherry fusion (top panel) or the parental strain without fusion used as a negative control (bottom panel) were inoculated from plates and grown 4 days in BM medium before red-fluorescence imaging. The overlay of red-fluorescence and bright field images is shown. Scale bar: 10 µm.
