# Supplementary material for: Prophage induction can facilitate the in vitro dispersal of multicellular Streptomyces structures
Source: PLoS Biol. 2024 Jul 25;22(7):e3002725. doi: 10.1371/journal.pbio.3002725 (PMC11302927; doi:10.1371/journal.pbio.3002725)
Supplement: S10 Fig — (PDF) [file pbio.3002725.s010.pdf]

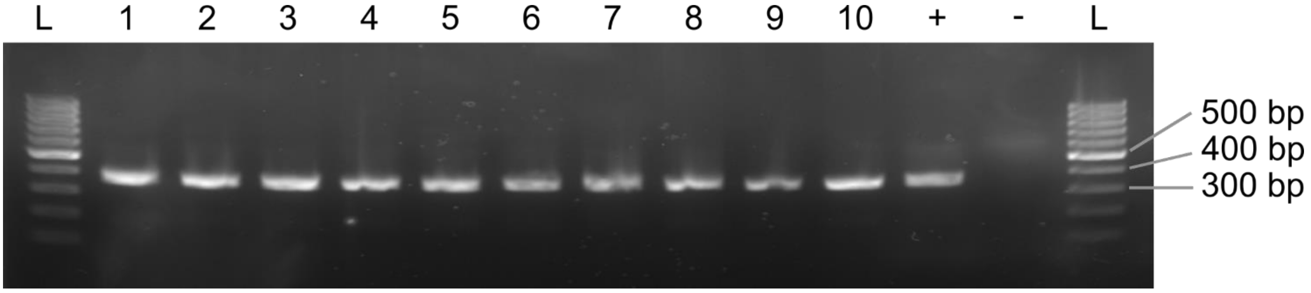

S10 Figure: Detection of Samy prophage within the genome of clones isolated on SFM plates after 4-days growth in BM liquid medium

Ten colonies (lines 1 to 10) from SFM plates collected after 4-days growth in BM medium were individually regrown in TSB medium. Genomic DNA was then extracted, and the presence of Samy was assessed via PCR amplification with SBM385 and SBM386 primers. PCR products were visualized on a 1.5% agarose gel stained with Atlas ClearSight DNA Stain. The parental strain *S. ambofaciens* ATCC 23877 and its Samy prophage-deleted derivative (Δ*Samy* #1) were used as positive ('+') and negative ('-') control, respectively. The expected PCR product size in the presence of Samy prophage within the strains is 348 bp. "L" indicates the GeneRuler™100 bp DNA ladder from NEB.
