# Supplementary material for: Prophage induction can facilitate the in vitro dispersal of multicellular Streptomyces structures
Source: PLoS Biol. 2024 Jul 25;22(7):e3002725. doi: 10.1371/journal.pbio.3002725 (PMC11302927; doi:10.1371/journal.pbio.3002725)
Supplement: S11 Fig — (PDF) [file pbio.3002725.s011.pdf]

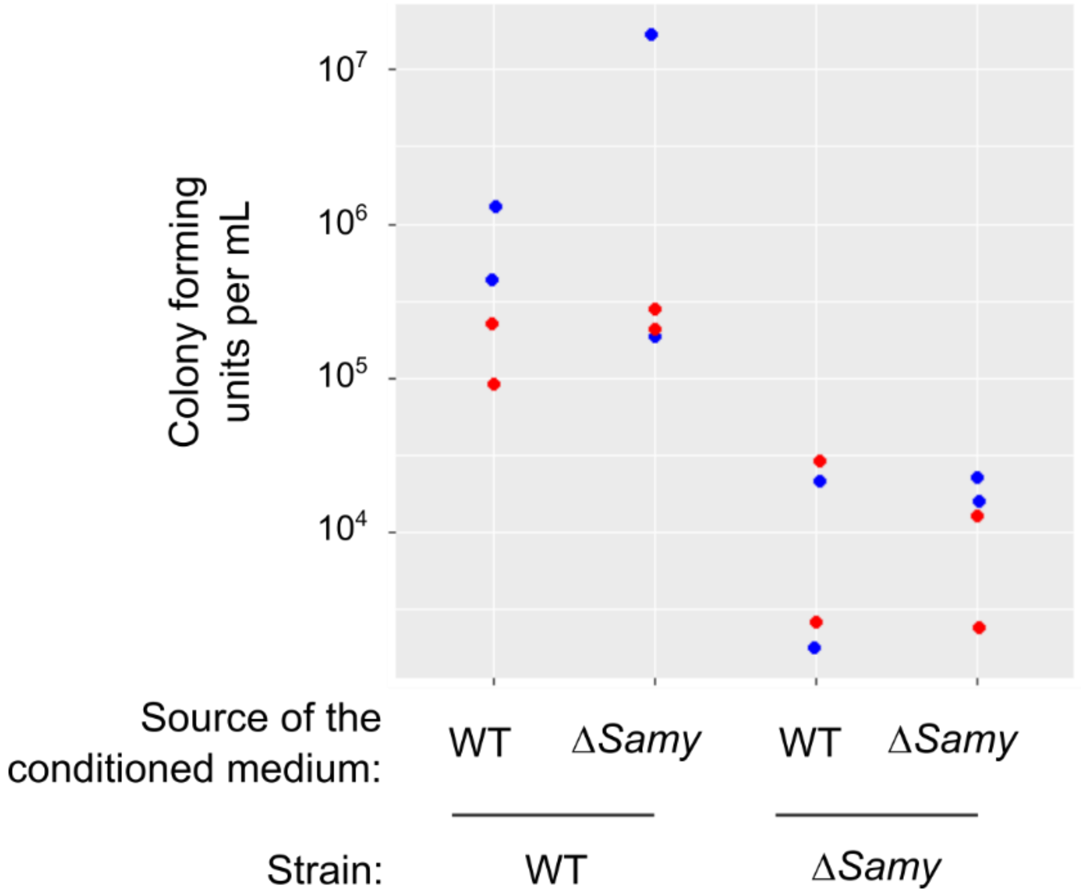

S11 Figure: Colony forming units after 4 days of growth in BM medium of *S. ambofaciens* ATCC 23877 reference strain and its isogenic Δ*Samy* clone #3 mutant supplemented with conditioned supernatants

S. ambofaciens ATCC 23877 WT strain or its isogenic mutant Δ*Samy* clone #3 (**S4 Table**) were grown for 24 h before supplementing the medium with filtered conditioned supernatant from a previous 4 days culture of the WT or Samy-deleted strain at ratios of 1:2 (blue) or 1:5 (red) of conditioned supernatant (0.2 µm filtered) per 24h-culture volumes. Then, bacteria were grown for an additional 3 days before counting. The results of 2 independent experiments are presented. The data and scripts underlying these panels can be found in **S1 Data** and **S2 Data**, respectively.
