# Supplementary material for: Prophage induction can facilitate the in vitro dispersal of multicellular Streptomyces structures
Source: PLoS Biol. 2024 Jul 25;22(7):e3002725. doi: 10.1371/journal.pbio.3002725 (PMC11302927; doi:10.1371/journal.pbio.3002725)
Supplement: S4 Table — (PDF) [file pbio.3002725.s015.pdf]

S4 Table: Strains used in this study*

Name	Main characteristics	Samy complete prophage	Reference
Strains used to perform Samy infection assays			
S. ambofaciens ATCC 23877	RP3486 strain deposited at the ATCC by RP - Sequenced genome (GCF_001267885.1_ASM 126788v1)	+	(1), genome sequence (2), Pernodet-Lautru's lab collection
S. ambofaciens DSM 40697	Tü13 strain deposited at the DSM collection - Sequenced genome (GCF_001632865.1, ASM163286v1)	-	(3), genome sequence (4), Pernodet-Lautru's lab collection
S. albidoflavus J1074/R2	Derivate from *S. albidoflavus* J1074	-	(5), Pernodet-Lautru's lab collection
S. coelicolor A(3)2	Type strain	-	(6), Pernodet-Lautru's lab collection
S. lividans 66 TK24	Streptomycin-resistant mutation (*str6)* genetic marker	-	(7), Pernodet-Lautru's lab collection
S. venezuelae ATCC 10712	*S. venezuelae* Ehrlich *et al.,* type strain isolated from soil in Venezuela	-	Pernodet-Lautru's lab collection
Other *Streptomyces ambofaciens* parental strains			
S. ambofaciens RP3486	Isolated from soil in France (Péronne, Somme) by RP	+	(1), Pernodet-Lautru's lab collection, initially obtained from RP
S. ambofaciens ETH 6703	Isolated from soil in Italy (Rome) by H. Zähner, collection from the ETH	-	(3), Pernodet-Lautru's lab collection
S. ambofaciens Tü13	Collection isolate of ETH 6703 from Tübingen university	-	(3), Pernodet-Lautru's lab collection
S. ambofaciens ETH 11317	Collection from the ETH, initially named *S. aureofaciens*	-	(3), (8), Pernodet-Lautru's lab collection
Derivates of *Streptomyces ambofaciens* RP3486			
S. ambofaciens NRRL 2420	*S. ambofaciens* Pinnert-Sindico (ATCC 15154) also named 'Rhone-Poulenc 1297-18-T2, obtained by mutagenesis (UV irradiation) of RP3486	+	Pernodet-Lautru's lab collection
S. ambofaciens JI3212	Derivate of ATCC 15154 maintained by the John Innes Centre (the strain	+	(9), Pernodet-Lautru's lab collection, initially

	harbors a mutation in pSAM2 compared to ATCC 15154)		obtained from the John Innes Institute collection
S. ambofaciens RP181110	Isolated after UV irradiation of RP3486	+	(9), Pernodet-Lautru's lab collection, initially obtained from RP
Derivates of *Streptomyces ambofaciens* ATCC 23877			
S. ambofaciens Δ*Samy* clone #1	Isolated after CRISPR-Cas9 engineering with a sgRNA targeting Samy integrase encoding gene. Harbors a large deletion (74.0 kb) compassing Samy and ≈ 12.7 kb downstream sequences (Δ6,589,490-6,663,535)	-	This study
S. ambofaciens Δ*Samy* clone #3	Isolated after CRISPR - Cas9 engineering with a sgRNA targeting Samy integrase encoding gene. Harbors a local deletion (≈ 13.2 kb) of the region surrounding Samy integrase gene and the remnant integrative element located upstream Samy (Δ6,581,646-6,594,874)	-	This study
S. ambofaciens Δ*Samy* clone #4	Isolated after CRISPR - Cas9 engineering with a sgRNA targeting Samy integrase encoding gene. Harbors a deletion (≈ 11.5 kb) of Samy integrase gene and all of the remnant integrative element located upstream Samy (Δ6,579,093-6,590,594)	-	This study

*Abbreviations: ATCC (American Type Culture Collection), DSM (*Deutsche Sammlung von Mikroorganismen*), ETH (*Eidgenössische Technischen Hochschule*, Zürich), RP (Rhône-Poulenc)

References:

1. Pinnert-Sindico S, Ninet L, Preud'homme J, Cosar C. A new antibiotic Spiramycin. Antibiotics annual. 1955 1954;p 724-727.

2. Thibessard A, Haas D, Gerbaud C, Aigle B, Lautru S, Pernodet JL, et al. Complete genome sequence of *Streptomyces ambofaciens* ATCC 23877, the spiramycin producer. J Biotechnol. 2015;214:117-8.

3. Hütter R. [Classification of the streptomycetes with special regard to the antibiotics formed from them]. Bibl Microbiol. 1967;6:1-382.

4. Thibessard A, Leblond P. Complete Genome Sequence of *Streptomyces ambofaciens* DSM 40697, a Paradigm for Genome Plasticity Studies. Genome Announc. 2016;4(3):e00470-16.

5. Rebets Y, Brötz E, Manderscheid N, Tokovenko B, Myronovskyi M, Metz P, et al. Insights into the Pamamycin Biosynthesis. Angew Chem Int Ed. 2015;54(7):2280-4.

6. Freeman RF, Bibb MJ, Hopwood DA. Chloramphenicol Acetyltransferase-independent Chloramphenicol Resistance in *Streptomyces coelicolor* A3(2). Journal of General Microbiology. 1977;98(2):453-65.

7. Hopwood DA, Kieser T, Wright HM, Bibb MJ. Plasmids, Recombination and Chromosome Mapping in *Streptomyces lividans* 66. Microbiology. 1983;129(7):2257-69.

8. Leblond P, Francou FX, Simonet JM, Decaris B. Pulsed-field gel electrophoresis analysis of the genome of *Streptomyces ambofaciens* strains. FEMS Microbiology Letters. 1990;72(1-2):79-88.

9. Pernodet JL, Simonet JM, Guérineau M. Plasmids in different strains of *Streptomyces ambofaciens*: free and integrated form of plasmid pSAM2. Molec Gen Genet. 1984;198(1):35-41.
