# Supplementary material for: Prophage induction can facilitate the in vitro dispersal of multicellular Streptomyces structures
Source: PLoS Biol. 2024 Jul 25;22(7):e3002725. doi: 10.1371/journal.pbio.3002725 (PMC11302927; doi:10.1371/journal.pbio.3002725)
Supplement: S5 Table — (PDF) [file pbio.3002725.s016.pdf]

S5 Table: Primers used in this study

Name	Sequence (5'-3')	Target
Quantitative PCR		
SBM399	CCGCTGACCTCGTCGTCTAC	Region 6,617,269-6,617,375 of *S. ambofaciens* ATCC 23877 chromosome corresponding to a Samy gene (SAMYPH_28/ SAM23877_RS29055) encoding a tail fiber protein
SBM400	GCCCTTGATGGTGTTCAGGA	
SBM446	CGAAGCCGCTCAGGCCAACC	Region 5,975,814-5,975,937 of *S. ambofaciens* ATCC 23877 chromosome containing part of the *srmS* (*srm40*, SAM23877_RS26595) *S. ambofaciens* ATCC 23877 gene encoding the regulator of the spiramycin BGC – This qPCR was used as a control to test the presence of host DNA after DNAse treatment.
SBM447	GCCCACCCGCACCATGAAGG	
Primers used to clone the sgRNA targeting Samy integrase		
SBM393-sgRNA –int2	CATG**CCATGG**ACACATCGACGATGGTAGGTGTTTTAGAGCTAGAAATAGC	*Nco*I-*Sna*BI sgRNA cloning site of pCRISPR-Cas9 vector from Tong *et al*.(1)
SBM67-scaffold-R	ACGCC**TACGTA**AAAAAAGCACCGACTCGGTGCC	
Primers used to assess the presence of the Samy prophage in the genomic DNA of bacteria surviving 4 days growth in BM medium		
SBM385	CGCCCGCGTAGGCAAACTGG	Region of 348 bp located within Samy prophage, from position 6,592,295 to 6,592,642 within *S. ambofaciens* ATCC 23877 chromosome
SBM386	GTGGAGCTGCCGTCCTCCTG	
Primers used to clone MCP-mCherry		
SBM548_MCP-UP-FOR	GGGGGTCTCTAACAATCAACGACCTGACGTTCCG	Region (920 bp) including the last non-stop codon of the gene encoding Samy major capsid protein
SBM549_MCP-UP-REV	CGCGGTCTCGGAGACCGCAATCACGTCCGC	
SBM550_cherry-FOR	GGGGGTCTCTTCTCCAAGGGGGAAGAGGACAACA	Synthetic mCherry encoding gene amplified in frame with MCP encoding gene (700 bp)
SBM551_cherry-REV	CGCGGTCTCGCTACTTGTAGAGTTCGTCCA	
SBM552_SpecR-FOR	GGGGGTCTCTGTAGCCACACTACCATCGGCGCTA	Spectinomycin resistance gene (1306 bp)
SBM553_SpecR-REV	ATTGGTCTCGGCGGCCGCGTGAAGGGCGTC	
SBM554_MCP_D-FOR	GGGGGTCTCTCCGCGTGAAGCACGCTGGGTCCGC	Region downstream MCP gene (882 bp)
SBM555_MCP_D-REV	CGCGGTCTCGCAGGAGGCCTAGACATGAGCTGGGCTACCA	
SBM375	GGTCTCCCCTGTGTGAAATTGTTATCC	Amplification of pOJ260 vector (2) to introduce BsaI sites
SBM376_BIS	AAACTATGACGGTCTCATGTTACGAATTCGATATCGCGC	

References:

1. Tong Y, Charusanti P, Zhang L, Weber T, Lee SY. CRISPR-Cas9 Based Engineering of Actinomycetal Genomes. ACS Synth Biol. 2015;4(9):1020- 9.

2. Bierman M, Logan R, O'Brien K, Seno ET, Rao RN, Schoner BE. Plasmid cloning vectors for the conjugal transfer of DNA from *Escherichia coli* to *Streptomyces spp*. Gene. 1992;116(1):43- 9.
