# Supplementary material for: Prophage induction can facilitate the in vitro dispersal of multicellular Streptomyces structures
Source: PLoS Biol. 2024 Jul 25;22(7):e3002725. doi: 10.1371/journal.pbio.3002725 (PMC11302927; doi:10.1371/journal.pbio.3002725)
Supplement: S6 Table — (PDF) [file pbio.3002725.s017.pdf]

S6 Table: Media used in this study

Name	Composition	Reference
Classical *Streptomyces* media		
HT (Hickey and Tresner)	1 g/L yeast extract, 1 g/L meat/beef extract (Difco™ Beef extract, ref 212610), 2 g/L bactotryptone, 10 g/L white dextrin, 0.02 g/L $CoCl_2$, with or without 20 g/L agar; pH 7.3	Adapted from (1), (2)
MM (Minimal Medium Mannitol)	0.5 g/L L-asparagine, 0.5 g/L K_2HPO_4, 0.2 g/L $MgSO_4$, 0.001 g/L $FeSO_4$, 0.5 % mannitol, 10 g/L agar; pH 7.0/7.2	(1)
MNB (MP Biomedicals™ Nutrient Broth)	5.0 g/L gelatin peptone, 3.0 g/L beef extract, pH: 6.9 +/- 0.1	Cat#091007 917
MP5 (medium of production n°5)	7 g/L yeast extract, 20.9 g/L MOPS, 5 g/L NaCl, 1 g/L $NaNO_3$, 36 mL/l glycerol; pH 7.5	(3)
ONA (Oxoid nutrient agar)	28g / L of Oxoid nutrient agar (corresponding to 1 g/L of meat/beef extract, 2g/L yeast extract, 5g/L pepton, 5 g/L NaCl, 15 g/L agar, pH7.4)	CM003 (Oxoid)
SAF	0.5 g/L yeast extract, 0.5 g/L meat/beef extract, 21 g/L MOPS, 5g/L glucose, 1 g/L enzymatic hydrolysate of casein, 20 g/L agar; pH 7.0	(4)
SNA (Soft Nutrient Agar)	5 g/L agar, 8 g/L MNB	(1)
SFM (Soy Flour-Mannitol)	20g/L organic soy flour, 20 g/L mannitol, 20 g/L agar	(1)
TSB (Tryptic soy broth)	Tryptic Soy Broth (30g/L)	BD™211825
Variants of the HT or MP5 media to generate the results presented in the Fig. 3		
HT - dextrin	1 g/L yeast extract, 1 g/L meat/beef extract, 2 g/L bactotryptone, 0.02 g/L $CoCl_2$; pH 7.3	This study
HT - meat	1 g/L yeast extract, 2 g/L bactotryptone, 10 g/L white dextrin, 0.02 g/L $CoCl_2$; pH 7.3	This study
HT – $CoCl_2$	1 g/L yeast extract, 1 g/L meat/beef extract, 2 g/L bactotryptone, 10 g/L white dextrin; pH 7.3	This study
HT - dextrin – meat - $CoCl_2$	1 g/L yeast extract, 2 g/L bactotryptone; pH 7.3	This study
HT - dextrin – meat	1 g/L yeast extract, 2 g/L bactotryptone, 0.02 g/L $CoCl_2$; pH 7.3	This study
BM (bacteriophage production medium)	1 g/L yeast extract, 1 g/L meat/beef extract, 2 g/L bactotryptone; pH 7.3	This study
BM + MOPS	1 g/L yeast extract, 1 g/L meat/beef extract, 2 g/L bactotryptone, 21 g/L MOPS; pH 7.3	This study
MP5 - MOPS	7 g/L yeast extract, 5 g/L NaCl, 1 g/L $NaNO_3$, 36 ml/l glycerol; pH 7.5	This study

References:

1. Kieser T. BMJ Buttner MJ, Chater KF, Hopwood DA. Practical *Streptomyces* genetics. John Innes Foundation, Norwich, United Kingdom. 2000.

2. Hickey RJ, Tresner HD. A cobalt-containing medium for sporulation of *Streptomyces* species. J Bacteriol. 1952;64(6):891-2.

3. Pernodet JL, Alegre MT, Blondelet-Rouault MH, Guerineau M. Resistance to spiramycin in *Streptomyces ambofaciens*, the producer organism, involves at least two different mechanisms. J Gen Microbiol. 1993;139(5):1003-11.

4. Sanchez L, Brana AF. Cell density influences antibiotic biosynthesis in *Streptomyces clavuligerus.* Microbiology. 1996;142 (Pt 5):1209-20.
