# Supplementary material for: Prophage induction can facilitate the in vitro dispersal of multicellular Streptomyces structures
Source: PLoS Biol. 2024 Jul 25;22(7):e3002725. doi: 10.1371/journal.pbio.3002725 (PMC11302927; doi:10.1371/journal.pbio.3002725)
Supplement: S2 Data — (HTML) [file pbio.3002725.s019.html]

Prophage induction can facilitate the in vitro dispersal of multicellular Streptomyces structures


# Prophage induction can facilitate the in vitro dispersal of multicellular Streptomyces structures

## Prophage induction can facilitate the in vitro dispersal of multicellular Streptomyces structures

- RNA-seq analysis using
  SARTools
- Figure 1A
- Figure 2B
- Figure 3A
- Figure 3B
- Figure 4A
- Figure 4D
- Figure 4F
- Figure 4G
- Figure S1
- Figure S3C
- Figure S4
- Figure S11

Stéphanie
Bury-Moné

2024-06-18

```
library(ggplot2)
library(readxl)
library("seqinr")
library("ggpubr")
library(mclust)
```

The RNA-seq data generated during this study have been deposited in
the NCBI Gene Expression Omnibus (GEO, https://www.ncbi.nlm.nih.gov/geo/) under the accession
code GSE232795 (https://www-ncbi-nlm-nih-gov.insb.bib.cnrs.fr/geo/query/acc.cgi?acc=GSE232795).

The numeric data used to generate the figures presented below are
available in S1 Data.

## RNA-seq analysis using SARTools

The reads were mapped on a genome harboring a single TIR.

```
#=========================================Create environments
conda create -n rnaseqEBAI -y

conda activate rnaseqEBAI
conda install -c bioconda fastqc
conda install -c bioconda star 
conda install -c bioconda subread
conda list -n rnaseqEBAI

#=========================================Data
#To obtain de file from SRA (here the SRA number is SRRXXXXX)
fastq-dump --split-files SRRXXXXX

#Quality control (here the fastq number/name is SRRXXXXX.fastq)
fastqc SRRXXXXX.fastq

#===========Prepare files
Prepare these directories:
- Fastq_files/ with the fastq files
- Genome/ with the reference genome
- Bam_files/

#=========================================To do the index (it will be in "Genome" directory):

STAR   --runMode genomeGenerate   --runThreadN 4   --genomeDir ./Genome   --genomeFastaFiles ./Genome/GCF_001267885.1_ASM126788v1_genomic_woTIR2.fna      --genomeSAindexNbases 4


#=========================================Mapping (example for HT replicate 1)

STAR --genomeDir ./Genome/ --runThreadN 4 --outFileNamePrefix ./Bam_files/WTHT_1. --readFilesIn ./Fastq_files/WTHT1_S1_all_R1_001.fastq ./Fastq_files/WTHT1_S1_all_R2_001.fastq --outSAMtype BAM SortedByCoordinate --alignIntronMax 1000 --alignMatesGapMax 10000 --limitBAMsortRAM 1259563133

#=========================================COUNTING
conda activate rnaseqEBAI
featureCounts -p -s 1 -t gene -g ID -a ./Annotation/GCF_001267885.1_ASM126788v1_genomic_CONGO_PHAGE_april2023.gff -T 4 -o ./Counts/WTHT_1_counts_S.txt ./Bam_files/WTHT_1.Aligned.sortedByCoord.out.bam
```

The RNA-seq analysis was performed using SARTools DESeq2-based R
pipeline. The statistical report of project corresponds to the S3
Data.

Reference: Varet H, Brillet-Gueguen L, Coppee JY, Dillies MA.
SARTools: A DESeq2- and EdgeR-Based R Pipeline for Comprehensive
Differential Analysis of RNA-Seq Data. PLoS One.
2016;11(6):e0157022.

```
#Installation of SARTools
install.packages("devtools")
devtools::install_github("PF2-pasteur-fr/SARTools", build_opts="--no-resave-data")

################################################################################
### R script to compare several conditions with the SARTools and DESeq2 packages
### Hugo Varet
### March 23rd, 2022
### designed to be executed with SARTools 1.8.1
################################################################################

################################################################################
###                parameters: to be modified by the user                    ###
################################################################################
rm(list=ls())                                        # remove all the objects from the R session

workDir <- "./"      # working directory for the R session

projectName <- "Samy phage OSMAC-RNAseq"                         # name of the project
author <- "Stéphanie Bury-Moné"                                # author of the statistical analysis/report

targetFile <- "target_SAM.txt"                           # path to the design/target file
rawDir <- "./Counts/"                                      # path to the directory containing raw counts files
featuresToRemove <- c("alignment_not_unique",        # names of the features to be removed
                      "ambiguous", "no_feature",     # (specific HTSeq-count information and rRNA for example)
                      "not_aligned", "too_low_aQual")# NULL if no feature to remove

varInt <- "Condition"                                    # factor of interest
condRef <- "MP24"                                      # reference biological condition
batch <- NULL                                        # blocking factor: NULL (default) or "batch" for example

fitType <- "parametric"                              # mean-variance relationship: "parametric" (default), "local" or "mean"
cooksCutoff <- TRUE                                  # TRUE/FALSE to perform the outliers detection (default is TRUE)
independentFiltering <- TRUE                         # TRUE/FALSE to perform independent filtering (default is TRUE)
alpha <- 0.05                                        # threshold of statistical significance
pAdjustMethod <- "BH"                                # p-value adjustment method: "BH" (default) or "BY"

typeTrans <- "VST"                                   # transformation for PCA/clustering: "VST" or "rlog"
locfunc <- "median"                                  # "median" (default) or "shorth" to estimate the size factors

#colors <- c(rainbow(4))
colors <- c("#f3c300", "#875692", "#f38400",         # vector of colors of each biological condition on the plots
            "#a1caf1", "#be0032", "#c2b280",
           "#848482", "#008856", "#e68fac",
            "#0067a5", "#f99379", "#604e97", "lightsalmon3")

forceCairoGraph <- FALSE

################################################################################
###                             running script                               ###
################################################################################
setwd(workDir)
library(SARTools)
if (forceCairoGraph) options(bitmapType="cairo")

# checking parameters
checkParameters.DESeq2(projectName=projectName,author=author,targetFile=targetFile,
                       rawDir=rawDir,featuresToRemove=featuresToRemove,varInt=varInt,
                       condRef=condRef,batch=batch,fitType=fitType,cooksCutoff=cooksCutoff,
                       independentFiltering=independentFiltering,alpha=alpha,pAdjustMethod=pAdjustMethod,
                       typeTrans=typeTrans,locfunc=locfunc,colors=colors)

# loading target file
target <- loadTargetFile(targetFile=targetFile, varInt=varInt, condRef=condRef, batch=batch)

# loading counts
counts <- loadCountData(target=target, rawDir=rawDir, featuresToRemove=featuresToRemove)

# description plots
majSequences <- descriptionPlots(counts=counts, group=target[,varInt], col=colors)

# analysis with DESeq2
out.DESeq2 <- run.DESeq2(counts=counts, target=target, varInt=varInt, batch=batch,
                         locfunc=locfunc, fitType=fitType, pAdjustMethod=pAdjustMethod,
                         cooksCutoff=cooksCutoff, independentFiltering=independentFiltering, alpha=alpha)

# PCA + clustering
exploreCounts(object=out.DESeq2$dds, group=target[,varInt], typeTrans=typeTrans, col=colors)

# summary of the analysis (boxplots, dispersions, diag size factors, export table, nDiffTotal, histograms, MA plot)
summaryResults <- summarizeResults.DESeq2(out.DESeq2, group=target[,varInt], col=colors,
                                          independentFiltering=independentFiltering,
                                          cooksCutoff=cooksCutoff, alpha=alpha)

# save image of the R session
save.image(file=paste0(projectName, ".RData"))

# generating HTML report
writeReport.DESeq2(target=target, counts=counts, out.DESeq2=out.DESeq2, summaryResults=summaryResults,
                   majSequences=majSequences, workDir=workDir, projectName=projectName, author=author,
                   targetFile=targetFile, rawDir=rawDir, featuresToRemove=featuresToRemove, varInt=varInt,
                   condRef=condRef, batch=batch, fitType=fitType, cooksCutoff=cooksCutoff,
                   independentFiltering=independentFiltering, alpha=alpha, pAdjustMethod=pAdjustMethod,
                   typeTrans=typeTrans, locfunc=locfunc, colors=colors)


#save homoscedastic data
countHOMOSC<-getVarianceStabilizedData(out.DESeq2$dds)

#Go into the tables and take pertinent set of analysis fol_change, adjusted p values
#Only for comparisons of interest

HTvsMM<-read.table("HTvsMM.complete.txt", header = T)
colnames(HTvsMM)[90]<-"FoldChange_HTvsMM"
colnames(HTvsMM)[91]<-"log2FoldChange_HTvsMM"
colnames(HTvsMM)[92]<-"stat_HTvsMM"
colnames(HTvsMM)[93]<-"pvalue_HTvsMM"
colnames(HTvsMM)[94]<-"padj_HTvsMM"
summary(HTvsMM)

NAGvsMM<-read.table("NAGvsMM.complete.txt", header = T)
colnames(NAGvsMM)[90]<-"FoldChange_NAGvsMM"
colnames(NAGvsMM)[91]<-"log2FoldChange_NAGvsMM"
colnames(NAGvsMM)[92]<-"stat_NAGvsMM"
colnames(NAGvsMM)[93]<-"pvalue_NAGvsMM"
colnames(NAGvsMM)[94]<-"padj_NAGvsMM"
summary(NAGvsMM)
dataset<-cbind(HTvsMM,NAGvsMM[,90:94])
summary(dataset)

ONAvsMM<-read.table("ONAvsMM.complete.txt", header = T)
colnames(ONAvsMM)[90]<-"FoldChange_ONAvsMM"
colnames(ONAvsMM)[91]<-"log2FoldChange_ONAvsMM"
colnames(ONAvsMM)[92]<-"stat_ONAvsMM"
colnames(ONAvsMM)[93]<-"pvalue_ONAvsMM"
colnames(ONAvsMM)[94]<-"padj_ONAvsMM"
summary(ONAvsMM)
dataset<-cbind(dataset,ONAvsMM[,90:94])
summary(dataset)

HTvsNAG<-read.table("HTvsNAG.complete.txt", header = T)
colnames(HTvsNAG)[90]<-"FoldChange_HTvsNAG"
colnames(HTvsNAG)[91]<-"log2FoldChange_HTvsNAG"
colnames(HTvsNAG)[92]<-"stat_HTvsNAG"
colnames(HTvsNAG)[93]<-"pvalue_HTvsNAG"
colnames(HTvsNAG)[94]<-"padj_HTvsNAG"
summary(HTvsNAG)
dataset<-cbind(dataset,HTvsNAG[,90:94])
summary(dataset)


HTvsONA<-read.table("HTvsONA.complete.txt", header = T)
colnames(HTvsONA)[90]<-"FoldChange_HTvsONA"
colnames(HTvsONA)[91]<-"log2FoldChange_HTvsONA"
colnames(HTvsONA)[92]<-"stat_HTvsONA"
colnames(HTvsONA)[93]<-"pvalue_HTvsONA"
colnames(HTvsONA)[94]<-"padj_HTvsONA"
summary(HTvsONA)
dataset<-cbind(dataset,HTvsONA[,90:94])
summary(dataset)

HTvsMP24<-read.table("HTvsMP24.complete.txt", header = T)
colnames(HTvsMP24)[90]<-"FoldChange_HTvsMP24"
colnames(HTvsMP24)[91]<-"log2FoldChange_HTvsMP24"
colnames(HTvsMP24)[92]<-"stat_HTvsMP24"
colnames(HTvsMP24)[93]<-"pvalue_HTvsMP24"
colnames(HTvsMP24)[94]<-"padj_HTvsMP24"
summary(HTvsMP24)
dataset<-cbind(dataset,HTvsMP24[,90:94])
summary(dataset)

dataset<-write.csv2(dataset, "dataset.csv", row.names = F)
```

## Figure 1A

The script requires to download from NCBI the fasta file of
*Streptomyces ambofaciens* ATCC 23877 genome:
“GCF\_001267885.1\_ASM126788v1\_genomic.fna”.

```
SAM_sequence <- read.fasta(file = "GCF_001267885.1_ASM126788v1_genomic.fna")
chr_seq <- SAM_sequence[[1]]

slidingwindowplot <- function(windowsize, inputseq)
{
  starts <- seq(1, length(inputseq)-windowsize, by = windowsize)
  n <- length(starts)
  chunkGCs <- numeric(n)
  for (i in 1:n) {
    chunk <- inputseq[starts[i]:(starts[i]+windowsize-1)]
    chunkGC <- GC(chunk)
    chunkGCs[i] <- chunkGC
  }
  plot(starts,chunkGCs,type="b",xlab="Nucleotide start position",ylab="GC content")
  starts
  chunkGCs
}

tab<-slidingwindowplot(50000, chr_seq)
```

```
write.csv2(tab,"GC_tab_SAM_ATCC23877_chromosome.csv")
```

## Figure 2B

```
Fig2B <- read_excel("Fig2B.xlsx")
ggplot(Fig2B, aes(x=HT_RPKLOG2, y=log2FoldChange_HTvsMP24, color=STATUS)) + 
  geom_point(size=1)+theme_minimal() +
  scale_color_manual(values=c("azure2","yellow","brown4", "chocolate1","cornflowerblue"))
```

## Figure 3A

```
Fig3A <- read_excel("Fig3A.xlsx")

ggplot(data=Fig3A, aes(x=Medium, y=ATB_cm))+geom_boxplot() + geom_jitter(width = 0.01)
```

```
ggplot(data=Fig3A, aes(x=Medium, y=pH))+geom_boxplot()+ scale_y_continuous(trans = 'log10')+ annotation_logticks(sides="l") + geom_jitter(width = 0.01)
```

```
ggplot(data=Fig3A, aes(x=Medium, y=phage_ml))+geom_boxplot()+ scale_y_continuous(trans = 'log10')+ annotation_logticks(sides="l") + geom_jitter(width = 0.01)
```

## Figure 3B

```
Fig3B <- read_excel("Fig3B.xlsx")

Fig3B$Time<-as.character(Fig3B$Time)
Fig3B$Experiment<-as.character(Fig3B$Experiment)

ggplot(data=Fig3B, aes(x=Time, y=phage_ml))+ annotation_logticks(sides="l") + scale_y_continuous(trans = 'log10')+ geom_jitter(width = 0.01) +
  geom_boxplot()+ geom_dotplot(aes(fill=Experiment), binaxis='y', stackdir='center', dotsize=0.5)
```

## Figure 4A

```
Fig4A <- read_excel("Fig4A.xlsx")

ggplot(data=Fig4A, aes(x=Time, y=pH, colour = CAT)) + geom_point(size = 3, aes(shape=Experiment))+
  scale_fill_manual(values = c("blue", "blue","darkgrey", "darkgrey"))+
  scale_color_manual(values = c("blue", "blue","darkgrey", "darkgrey")) + theme_minimal() + facet_grid(cols = vars(Medium))
```

```
ggplot(data=Fig4A, aes(x=Time, y=OD, colour = CAT)) + geom_point(size = 3, aes(shape=Experiment))+ scale_y_continuous(trans = 'log10')+ annotation_logticks(sides="l")+
  scale_fill_manual(values = c("blue", "blue","darkgrey", "darkgrey"))+
  scale_color_manual(values = c("blue", "blue","darkgrey", "darkgrey")) + theme_minimal() + facet_grid(cols = vars(Medium))
```

## Figure 4D

```
Fig4D <- read_excel("Fig4D.xlsx")

ggplot(data=Fig4D, aes(x=CAT, y=CFU_ml, colour = Strain)) + geom_boxplot() + scale_y_continuous(trans = 'log10')+
  scale_color_manual(values = c("blue", "grey","red","yellow","violet"))+ geom_jitter(width=0.02)
```

```
phage_BM<-subset(Fig4D, Fig4D$CAT=="phage")
dsm_BM<-subset(Fig4D, Fig4D$CAT=="DSM_40697")
atcc_BM<-subset(Fig4D, Fig4D$CAT=="ATCC_23877")
wilcox.test(dsm_BM$CFU_ml, atcc_BM$CFU_ml)
```

```
## 
##  Wilcoxon rank sum exact test
## 
## data:  dsm_BM$CFU_ml and atcc_BM$CFU_ml
## W = 0, p-value = 4.114e-05
## alternative hypothesis: true location shift is not equal to 0
```

```
wilcox.test(phage_BM$CFU_ml, atcc_BM$CFU_ml)
```

```
## 
##  Wilcoxon rank sum exact test
## 
## data:  phage_BM$CFU_ml and atcc_BM$CFU_ml
## W = 0, p-value = 2.447e-06
## alternative hypothesis: true location shift is not equal to 0
```

```
wilcox.test(phage_BM$CFU_ml, dsm_BM$CFU_ml)
```

```
## 
##  Wilcoxon rank sum test with continuity correction
## 
## data:  phage_BM$CFU_ml and dsm_BM$CFU_ml
## W = 53, p-value = 0.5493
## alternative hypothesis: true location shift is not equal to 0
```

## Figure 4F

```
Fig4F <- read_excel("Fig4F.xlsx")

ggscatter(Fig4F, x = "log_Final_phage_per_µl", y = "log_CFU_per_ml", shape = "SAMY",  add = "reg.line", conf.int = TRUE, 
          cor.coef = TRUE, cor.method = "spearman",
          xlab = "Viral titer (phage per µl, log10)", ylab = "CFU per ml (log10)")+ scale_y_continuous(trans = 'log10')+ annotation_logticks(sides="l")
```

```
ggscatter(Fig4F, x = "log_Final_phage_per_µl", y = "log_CFU_per_ml", shape = "SAMY", color = "STRAIN",
          xlab = "Viral titer (phage per µl, log10)", ylab = "CFU per ml (log10)")+ scale_y_continuous(trans = 'log10')+ annotation_logticks(sides="l")
```

## Figure 4G

```
Fig4G <- read_excel("Fig4G.xlsx")

ggplot(data=Fig4G, aes(x=CAT, y=ATB, colour = Strain)) + geom_boxplot() + scale_y_continuous(trans = 'log10')+
  scale_color_manual(values = c("blue", "grey","red","yellow","violet"))+ geom_jitter(width=0.02)
```

```
ggplot(data=Fig4G, aes(x=CAT, y=ATB, colour = CAT)) + geom_boxplot() + scale_y_continuous(trans = 'log10')+
  scale_color_manual(values = c("blue", "red"))+ geom_jitter(width=0.02)
```

```
phage_BM<-subset(Fig4G, Fig4G$CAT=="phage_BM")
atcc_BM<-subset(Fig4G, Fig4G$CAT=="ATCC_23877_BM")
wilcox.test(phage_BM$ATB, atcc_BM$ATB)
```

```
## 
##  Wilcoxon rank sum test with continuity correction
## 
## data:  phage_BM$ATB and atcc_BM$ATB
## W = 27, p-value = 0.7425
## alternative hypothesis: true location shift is not equal to 0
```

## Figure S1

```
Table_S1 <- read_excel("Table_S1.xlsx")

Table_S1$GC_percent<-as.numeric(Table_S1$GC_percent)
ggplot(Table_S1, aes(x=GC_percent)) + geom_histogram(binwidth=1, aes(y=..density..), colour="black", fill="black")+ geom_vline(aes(xintercept=66.5275),
                                                                                                                                color="blue", linetype="dashed", size=1)+ geom_vline(aes(xintercept=median(GC_percent)),
                                                                                                                                                                                     color="red", linetype="dashed", size=1)+
  geom_density(alpha=.2, fill="#FF6666") + geom_vline(aes(xintercept=mean(GC_percent)),
                                                      color="violet", linetype="dashed", size=1)
```

```
ggplot(Table_S1, aes(x=Genome_Length_bp)) + geom_histogram(binwidth=1000, aes(y=..density..), colour="black", fill="black")+ geom_vline(aes(xintercept=61080),
                                                                                                                                     color="blue", linetype="dashed", size=1)+ geom_vline(aes(xintercept=median(Genome_Length_bp)),
                                                                                                                                                                                          color="red", linetype="dashed",size=1)+
  geom_density(alpha=.2, fill="#FF6666") + geom_vline(aes(xintercept=mean(Genome_Length_bp)),
                                                      color="violet", linetype="dashed", size=1)
```

## Figure S3C

```
FigS3C <- read_excel("FigS3C.xlsx")
boxplot(FigS3C$MP24_RPKLOG2)
```

## Figure S4

The ‘mclust’ R package was used to identify the gene populations of
Samy prophage transcriptomes, via a Gaussian finite mixture model fitted
by the expectation-maximization algorithm as followed. The script gives
the example for one condition (“MP24”). It was run for all conditions
the same way.

Reference: Scrucca L, Fop M, Murphy T Brendan, Raftery A E. mclust 5:
Clustering, Classification and Density Estimation Using Gaussian Finite
Mixture Models. The R Journal. 2016;8(1):289.

```
library(mclust)
Table_S2 <- read_excel("Table_S2.xlsx")
PROPHAGE<-subset(Table_S2, Table_S2$SAMYPH_annotation!="NA")

X<-PROPHAGE$MP24_RPKLOG2#Enter in a vector named X the log2 values of the normalized reads per Kb corresponding to each gene of the  prophage in the condition of interest (here "MP24") 
#vignette("mclust")
#Gaussian finite mixture model fitted by EM (Expectation-Maximization) algorithm 
BIC<-mclustBIC(X)
plot(BIC)
```

```
summary(BIC)
```

```
## Best BIC values:
##                V,2          E,2         V,3
## BIC      -480.8642 -481.5313324 -484.222511
## BIC diff    0.0000   -0.6671383   -3.358317
```

```
mod1<-Mclust(data=X, x = BIC)
summary(mod1, parameters = TRUE)
```

```
## ---------------------------------------------------- 
## Gaussian finite mixture model fitted by EM algorithm 
## ---------------------------------------------------- 
## 
## Mclust V (univariate, unequal variance) model with 2 components: 
## 
##  log-likelihood   n df       BIC       ICL
##       -228.8697 102  5 -480.8642 -505.0091
## 
## Clustering table:
##  1  2 
## 76 26 
## 
## Mixing probabilities:
##        1        2 
## 0.716821 0.283179 
## 
## Means:
##        1        2 
## 3.094937 7.572326 
## 
## Variances:
##        1        2 
## 1.663845 9.240840
```

```
plot(mod1, what="classification")
```

```
plot(mod1, what = "uncertainty")
```

```
table(mod1$classification)
```

```
## 
##  1  2 
## 76 26
```

```
#Annotation of Samy genes regarding the classification
PROPHAGE$EM_classif_MP24<-mod1$classification
```

Script used to generate FigS4

```
FigS4 <- read_excel("FigS4.xlsx")

FigS4$Condition <- factor(FigS4$Condition,      # Reordering group factor levels
                         levels = c("MP24", "MP30", "MP36", "MP48", "MP72", "Y24", "Ycongo24", "Y48", "SAF", "MM", "NAG", "ONA","HT"))

p<-ggplot(FigS4, aes(x=RPKLOG2, color = EM_classif, fill = EM_classif)) + 
  geom_histogram(alpha=0.5, position="identity")+
  labs(x="Sense transcription (DeSeq2reads per kb, log2)", y = "Counts")+theme_minimal()
facet(p, facet.by = "Condition")
```

## Figure S11

```
FigS11 <- read_excel("FigS11.xlsx")

ggplot(data=FigS11, aes(x=Condition, y=Value, colour = Ratio)) + scale_y_continuous(trans = 'log10')+
  scale_fill_manual(values = c("blue","red"))+
  scale_color_manual(values = c("blue","red"))+ geom_jitter(width=0.02)
```
