# Supplementary material for: Prophage induction can facilitate the in vitro dispersal of multicellular Streptomyces structures
Source: PLoS Biol. 2024 Jul 25;22(7):e3002725. doi: 10.1371/journal.pbio.3002725 (PMC11302927; doi:10.1371/journal.pbio.3002725)
Supplement: S3 Data — (HTML) [file pbio.3002725.s020.html]

 

 

 

 
 
 


 

 

 Statistical report of project Samy phage OSMAC-RNAseq: pairwise comparison(s) of conditions with DESeq2 

 
 
 
 
 
 
 
 
 
 
 
 
 
 
 
 

 

 
 


 


 


 

 

 


 


 

 


 


 
 
 
 
 
 

 


 


 Statistical report of project Samy phage
OSMAC-RNAseq: pairwise comparison(s) of conditions with DESeq2 
 Stéphanie Bury-Moné 
 2023-04-24 

 


 The SARTools R package which generated this report has been
developped at PF2 - Institut Pasteur by M.-A. Dillies and H. Varet ( hugo.varet@pasteur.fr ). Thanks to cite H. Varet, L.
Brillet-Guéguen, J.-Y. Coppee and M.-A. Dillies,  SARTools: A DESeq2-
and EdgeR-Based R Pipeline for Comprehensive Differential Analysis of
RNA-Seq Data , PLoS One, 2016, doi:  http://dx.doi.org/10.1371/journal.pone.0157022  when
using this tool for any analysis published. 
 
  1  Introduction 
 The analyses reported in this document are part of the Samy phage
OSMAC-RNAseq project. The aim is to find features that are
differentially expressed between MP24, MP30, MP36, MP48, MP72, Ycongo24,
Y24, Y48, MM, SAF, NAG, ONA and HT. The statistical analysis process
includes data normalization, graphical exploration of raw and normalized
data, test for differential expression for each feature between the
conditions, raw p-value adjustment and export of lists of features
having a significant differential expression between the conditions. 
 The analysis is performed using the R software  [1] , Bioconductor  [2]  packages including DESeq2  [3,4]  and the SARTools package developed at PF2
- Institut Pasteur. Normalization and differential analysis are carried
out according to the DESeq2 model and package. This report comes with
additional tab-delimited text files that contain lists of differentially
expressed features. 
 For more details about the DESeq2 methodology, please refer to its
related publications  [3,4] . 
 
 
  2  Description of raw
data 
 The count data files and associated biological conditions are listed
in the following table. 
 
 
Table 1: Data files and associated biological conditions.
 
 
 
 
Name
 
 
File
 
 
Condition
 
 
Batch
 
 
 
 
 
 
MP24a
 
 
WTMP5_24_1_counts_S.txt
 
 
MP24
 
 
RNA-3
 
 
 
 
MP24b
 
 
WTMP5_24_2_counts_S.txt
 
 
MP24
 
 
RNA-3
 
 
 
 
MP24c
 
 
WTMP5_24_3_counts_S.txt
 
 
MP24
 
 
RNA-3
 
 
 
 
MP30a
 
 
WTMP5_30_1_counts_S.txt
 
 
MP30
 
 
RNA-3
 
 
 
 
MP30b
 
 
WTMP5_30_2_counts_S.txt
 
 
MP30
 
 
RNA-3
 
 
 
 
MP30c
 
 
WTMP5_30_3_counts_S.txt
 
 
MP30
 
 
RNA-3
 
 
 
 
MP36a
 
 
WTMP5_36_1_counts_S.txt
 
 
MP36
 
 
RNA-3
 
 
 
 
MP36b
 
 
WTMP5_36_2_counts_S.txt
 
 
MP36
 
 
RNA-3
 
 
 
 
MP48a
 
 
WTMP5_48_1_counts_S.txt
 
 
MP48
 
 
RNA-3
 
 
 
 
MP48b
 
 
WTMP5_48_2_counts_S.txt
 
 
MP48
 
 
RNA-3
 
 
 
 
MP48c
 
 
WTMP5_48_3_counts_S.txt
 
 
MP48
 
 
RNA-3
 
 
 
 
MP72a
 
 
WTMP5_72_1_counts_S.txt
 
 
MP72
 
 
RNA-2
 
 
 
 
MP72b
 
 
WTMP5_72_2_counts_S.txt
 
 
MP72
 
 
RNA-2
 
 
 
 
MP72c
 
 
WTMP5_72_3_counts_S.txt
 
 
MP72
 
 
RNA-2
 
 
 
 
MP72d
 
 
WTMP5_72_4_counts_S.txt
 
 
MP72
 
 
RNA-2
 
 
 
 
Ycongo24a
 
 
WTYEMcongo_24_1_counts_S.txt
 
 
Ycongo24
 
 
RNA-3
 
 
 
 
Ycongo24b
 
 
WTYEMcongo_24_2_counts_S.txt
 
 
Ycongo24
 
 
RNA-3
 
 
 
 
Ycongo24c
 
 
WTYEMcongo_24_3_counts_S.txt
 
 
Ycongo24
 
 
RNA-3
 
 
 
 
Y24a
 
 
WTYEM_24_1_counts_S.txt
 
 
Y24
 
 
RNA-3
 
 
 
 
Y24b
 
 
WTYEM_24_2_counts_S.txt
 
 
Y24
 
 
RNA-3
 
 
 
 
Y24c
 
 
WTYEM_24_3_counts_S.txt
 
 
Y24
 
 
RNA-3
 
 
 
 
Y48a
 
 
WTYEM_48_1_counts_S.txt
 
 
Y48
 
 
RNA-3
 
 
 
 
Y48b
 
 
WTYEM_48_2_counts_S.txt
 
 
Y48
 
 
RNA-3
 
 
 
 
Y48c
 
 
WTYEM_48_3_counts_S.txt
 
 
Y48
 
 
RNA-3
 
 
 
 
MMa
 
 
WTMM_1_counts_S.txt
 
 
MM
 
 
RNA-2
 
 
 
 
MMb
 
 
WTMM_2_counts_S.txt
 
 
MM
 
 
RNA-2
 
 
 
 
SAFa
 
 
SAF_1_counts_S.txt
 
 
SAF
 
 
RNA-2
 
 
 
 
SAFb
 
 
SAF_2_counts_S.txt
 
 
SAF
 
 
RNA-2
 
 
 
 
SAFc
 
 
SAF_3_counts_S.txt
 
 
SAF
 
 
RNA-2
 
 
 
 
NAGa
 
 
WTNAG_1_counts_S.txt
 
 
NAG
 
 
RNA-2
 
 
 
 
NAGb
 
 
WTNAG_3_counts_S.txt
 
 
NAG
 
 
RNA-2
 
 
 
 
ONAa
 
 
WTONA_1_counts_S.txt
 
 
ONA
 
 
RNA-2
 
 
 
 
ONAb
 
 
WTONA_2_counts_S.txt
 
 
ONA
 
 
RNA-2
 
 
 
 
ONAc
 
 
WTONA_3_counts_S.txt
 
 
ONA
 
 
RNA-2
 
 
 
 
HTa
 
 
WTHT_1_counts_S.txt
 
 
HT
 
 
RNA-2
 
 
 
 
HTb
 
 
WTHT_2_counts_S.txt
 
 
HT
 
 
RNA-2
 
 
 
 
HTc
 
 
WTHT_3_counts_S.txt
 
 
HT
 
 
RNA-2
 
 
 
 
 After loading the data we first have a look at the raw data table
itself. The data table contains one row per annotated feature and one
column per sequenced sample. Row names of this table are feature IDs
(unique identifiers). The table contains raw count values representing
the number of reads that map onto the features. For this project, there
are 7534 features in the count data table. 
 
 
Table 2: Partial view of the count data table.
 
 
 
 
 
 
MP24a
 
 
MP24b
 
 
MP24c
 
 
MP30a
 
 
MP30b
 
 
MP30c
 
 
MP36a
 
 
MP36b
 
 
MP48a
 
 
MP48b
 
 
 
 
 
 
gene-SAM23877_6078
 
 
0
 
 
0
 
 
0
 
 
0
 
 
0
 
 
1
 
 
0
 
 
0
 
 
1
 
 
0
 
 
 
 
gene-SAM23877_6088
 
 
9
 
 
6
 
 
15
 
 
0
 
 
4
 
 
6
 
 
4
 
 
6
 
 
18
 
 
24
 
 
 
 
gene-SAM23877_6091
 
 
316
 
 
216
 
 
370
 
 
253
 
 
407
 
 
224
 
 
191
 
 
171
 
 
209
 
 
245
 
 
 
 
gene-SAM23877_6105
 
 
4
 
 
3
 
 
5
 
 
1
 
 
4
 
 
20
 
 
18
 
 
6
 
 
4
 
 
14
 
 
 
 
gene-SAM23877_6126
 
 
10
 
 
3
 
 
10
 
 
3
 
 
6
 
 
4
 
 
1
 
 
1
 
 
1
 
 
3
 
 
 
 
gene-SAM23877_6132
 
 
2
 
 
2
 
 
1
 
 
2
 
 
1
 
 
3
 
 
1
 
 
1
 
 
2
 
 
0
 
 
 
 
 Looking at the summary of the count table provides a basic
description of these raw counts (min and max values, median, etc). 
 
 
Table 3: Summary of the raw counts.
 
 
 
 
 
 
Min.
 
 
1st Qu.
 
 
Median
 
 
Mean
 
 
3rd Qu.
 
 
Max.
 
 
 
 
 
 
MP24a
 
 
0
 
 
44
 
 
290
 
 
3520
 
 
1534
 
 
3026484
 
 
 
 
MP24b
 
 
0
 
 
37
 
 
242
 
 
3101
 
 
1343
 
 
3032883
 
 
 
 
MP24c
 
 
0
 
 
33
 
 
205
 
 
2337
 
 
962
 
 
4092448
 
 
 
 
MP30a
 
 
0
 
 
23
 
 
141
 
 
1983
 
 
698
 
 
5100683
 
 
 
 
MP30b
 
 
0
 
 
20
 
 
123
 
 
2329
 
 
599
 
 
8990862
 
 
 
 
MP30c
 
 
0
 
 
31
 
 
176
 
 
2165
 
 
733
 
 
6811855
 
 
 
 
MP36a
 
 
0
 
 
25
 
 
163
 
 
1985
 
 
732
 
 
6451364
 
 
 
 
MP36b
 
 
0
 
 
15
 
 
90
 
 
1542
 
 
396
 
 
6159407
 
 
 
 
MP48a
 
 
0
 
 
44
 
 
211
 
 
2537
 
 
984
 
 
3345901
 
 
 
 
MP48b
 
 
0
 
 
48
 
 
235
 
 
2573
 
 
1031
 
 
5619211
 
 
 
 
MP48c
 
 
0
 
 
48
 
 
256
 
 
2175
 
 
1128
 
 
2763736
 
 
 
 
MP72a
 
 
0
 
 
11
 
 
53
 
 
1392
 
 
250
 
 
6053064
 
 
 
 
MP72b
 
 
0
 
 
16
 
 
79
 
 
1117
 
 
386
 
 
2048043
 
 
 
 
MP72c
 
 
0
 
 
10
 
 
54
 
 
980
 
 
262
 
 
2791148
 
 
 
 
MP72d
 
 
0
 
 
10
 
 
56
 
 
1043
 
 
285
 
 
2391622
 
 
 
 
Ycongo24a
 
 
0
 
 
24
 
 
159
 
 
2711
 
 
852
 
 
6811646
 
 
 
 
Ycongo24b
 
 
0
 
 
18
 
 
123
 
 
1683
 
 
649
 
 
3655746
 
 
 
 
Ycongo24c
 
 
0
 
 
31
 
 
203
 
 
2789
 
 
1079
 
 
3326219
 
 
 
 
Y24a
 
 
0
 
 
26
 
 
187
 
 
2524
 
 
1097
 
 
3106591
 
 
 
 
Y24b
 
 
0
 
 
36
 
 
239
 
 
2474
 
 
1262
 
 
2470345
 
 
 
 
Y24c
 
 
0
 
 
17
 
 
107
 
 
3179
 
 
543
 
 
12537082
 
 
 
 
Y48a
 
 
0
 
 
37
 
 
175
 
 
1608
 
 
748
 
 
2315818
 
 
 
 
Y48b
 
 
0
 
 
54
 
 
265
 
 
2233
 
 
1106
 
 
3463663
 
 
 
 
Y48c
 
 
0
 
 
47
 
 
224
 
 
2213
 
 
959
 
 
2938187
 
 
 
 
MMa
 
 
0
 
 
14
 
 
74
 
 
1147
 
 
374
 
 
2196922
 
 
 
 
MMb
 
 
0
 
 
21
 
 
107
 
 
1654
 
 
510
 
 
3691781
 
 
 
 
SAFa
 
 
0
 
 
19
 
 
97
 
 
997
 
 
444
 
 
1366659
 
 
 
 
SAFb
 
 
0
 
 
17
 
 
86
 
 
1070
 
 
414
 
 
1438974
 
 
 
 
SAFc
 
 
0
 
 
22
 
 
109
 
 
1141
 
 
486
 
 
1352203
 
 
 
 
NAGa
 
 
0
 
 
16
 
 
90
 
 
1093
 
 
427
 
 
976021
 
 
 
 
NAGb
 
 
0
 
 
20
 
 
109
 
 
1352
 
 
513
 
 
2554803
 
 
 
 
ONAa
 
 
0
 
 
15
 
 
79
 
 
947
 
 
382
 
 
1509274
 
 
 
 
ONAb
 
 
0
 
 
15
 
 
78
 
 
1037
 
 
381
 
 
1373148
 
 
 
 
ONAc
 
 
0
 
 
17
 
 
84
 
 
1049
 
 
407
 
 
1481612
 
 
 
 
HTa
 
 
0
 
 
16
 
 
71
 
 
707
 
 
299
 
 
1279775
 
 
 
 
HTb
 
 
0
 
 
20
 
 
91
 
 
1111
 
 
413
 
 
1928591
 
 
 
 
HTc
 
 
0
 
 
24
 
 
108
 
 
1085
 
 
466
 
 
1638213
 
 
 
 
 Figure 1 shows the total number of mapped and counted reads for each
sample. We expect total read counts to be similar within conditions,
they may be different across conditions. Total counts sometimes vary
widely between replicates. This may happen for several reasons,
including: 
 
 different rRNA contamination levels between samples (even between
biological replicates); 
 slight differences between library concentrations, since they may be
difficult to measure with high precision. 
 
 
 
 
 Figure 1: Number of mapped reads per sample. Colors
refer to the biological condition of the sample. 
 
 
 Figure 2 shows the percentage of features with no read count in each
sample. We expect this percentage to be similar within conditions.
Features with null read counts in the 37 samples are left in the data
but are not taken into account for the analysis with DESeq2. Here, 203
features (2.69%) are in this situation (dashed line). Results for those
features (fold-change and p-values) are set to NA in the results
files. 
 
 
 
 Figure 2: Percentage of features with null read
counts in each sample. 
 
 
 Figure 3 shows the distribution of read counts for each sample (on a
log scale to improve readability). Again we expect replicates to have
similar distributions. In addition, this figure shows if read counts are
preferably low, medium or high. This depends on the organisms as well as
the biological conditions under consideration. 
 
 
 
 Figure 3: Density distribution of read counts. 
 
 
 It may happen that one or a few features capture a high proportion of
reads (up to 20% or more). This phenomenon should not influence the
normalization process. The DESeq2 normalization has proved to be robust
to this situation [Dillies, 2012]. Anyway, we expect these high count
features to be the same across replicates. They are not necessarily the
same across conditions. Figure 4 and table 4 illustrate the possible
presence of such high count features in the data set. 
 
 
 
 Figure 4: Percentage of reads associated with the
sequence having the highest count (provided in each box on the graph)
for each sample. 
 
 
 
 
Table 4: Percentage of reads associated with the sequences having the
highest counts.
 
 
 
 
 
 
gene-SAM23877_RS36805
 
 
gene-SAM23877_RS36150
 
 
gene-SAM23877_RS09750
 
 
gene-SAM23877_RS21195
 
 
gene-SAM23877_RS20495
 
 
gene-SAM23877_RS21615
 
 
gene-SAM23877_RS11090
 
 
gene-SAM23877_RS15400
 
 
gene-SAM23877_RS22575
 
 
gene-SAM23877_RS11470
 
 
 
 
 
 
MP24a
 
 
11.41
 
 
2.45
 
 
1.55
 
 
1.29
 
 
0.74
 
 
0.57
 
 
0.00
 
 
0.02
 
 
0.02
 
 
0.08
 
 
 
 
MP24b
 
 
12.98
 
 
3.48
 
 
1.43
 
 
1.15
 
 
0.72
 
 
0.49
 
 
0.00
 
 
0.02
 
 
0.09
 
 
0.10
 
 
 
 
MP24c
 
 
23.24
 
 
4.99
 
 
0.88
 
 
0.96
 
 
0.94
 
 
0.48
 
 
0.00
 
 
0.01
 
 
0.06
 
 
0.10
 
 
 
 
MP30a
 
 
34.14
 
 
8.82
 
 
0.79
 
 
0.66
 
 
1.03
 
 
0.31
 
 
0.00
 
 
0.08
 
 
0.09
 
 
0.10
 
 
 
 
MP30b
 
 
51.23
 
 
11.25
 
 
0.46
 
 
0.32
 
 
0.97
 
 
0.20
 
 
0.00
 
 
0.02
 
 
0.04
 
 
0.05
 
 
 
 
MP30c
 
 
41.76
 
 
8.53
 
 
0.52
 
 
0.35
 
 
0.46
 
 
0.38
 
 
0.00
 
 
0.00
 
 
0.06
 
 
0.06
 
 
 
 
MP36a
 
 
43.14
 
 
4.09
 
 
0.40
 
 
0.19
 
 
0.48
 
 
0.55
 
 
0.26
 
 
0.08
 
 
0.16
 
 
0.13
 
 
 
 
MP36b
 
 
53.03
 
 
9.32
 
 
0.27
 
 
0.11
 
 
0.29
 
 
0.26
 
 
0.56
 
 
0.05
 
 
0.20
 
 
0.08
 
 
 
 
MP48a
 
 
17.51
 
 
1.10
 
 
0.42
 
 
0.15
 
 
0.26
 
 
0.03
 
 
0.02
 
 
1.27
 
 
0.77
 
 
0.36
 
 
 
 
MP48b
 
 
28.98
 
 
1.87
 
 
0.40
 
 
0.10
 
 
0.35
 
 
0.03
 
 
0.06
 
 
0.63
 
 
0.46
 
 
0.33
 
 
 
 
MP48c
 
 
16.86
 
 
1.80
 
 
0.49
 
 
0.22
 
 
0.39
 
 
0.25
 
 
0.28
 
 
0.12
 
 
1.02
 
 
0.16
 
 
 
 
MP72a
 
 
57.73
 
 
7.79
 
 
0.22
 
 
0.02
 
 
0.07
 
 
0.10
 
 
0.00
 
 
0.16
 
 
0.05
 
 
0.65
 
 
 
 
MP72b
 
 
24.33
 
 
2.14
 
 
0.54
 
 
0.05
 
 
0.08
 
 
0.20
 
 
0.00
 
 
0.77
 
 
0.17
 
 
1.72
 
 
 
 
MP72c
 
 
37.82
 
 
3.40
 
 
0.48
 
 
0.04
 
 
0.09
 
 
0.18
 
 
0.00
 
 
0.60
 
 
0.11
 
 
1.27
 
 
 
 
MP72d
 
 
30.44
 
 
2.32
 
 
0.38
 
 
0.04
 
 
0.06
 
 
0.24
 
 
0.00
 
 
0.90
 
 
0.23
 
 
2.02
 
 
 
 
Ycongo24a
 
 
33.35
 
 
8.09
 
 
0.81
 
 
0.65
 
 
1.21
 
 
0.54
 
 
0.00
 
 
0.01
 
 
0.02
 
 
0.06
 
 
 
 
Ycongo24b
 
 
28.83
 
 
7.12
 
 
0.85
 
 
0.62
 
 
1.31
 
 
0.40
 
 
0.00
 
 
0.01
 
 
0.04
 
 
0.08
 
 
 
 
Ycongo24c
 
 
15.83
 
 
6.93
 
 
1.66
 
 
1.29
 
 
0.74
 
 
0.60
 
 
0.00
 
 
0.03
 
 
0.02
 
 
0.07
 
 
 
 
Y24a
 
 
16.34
 
 
3.96
 
 
1.39
 
 
0.99
 
 
0.75
 
 
0.48
 
 
0.00
 
 
0.03
 
 
0.05
 
 
0.09
 
 
 
 
Y24b
 
 
13.25
 
 
4.34
 
 
1.25
 
 
0.97
 
 
1.19
 
 
0.47
 
 
0.00
 
 
0.01
 
 
0.04
 
 
0.12
 
 
 
 
Y24c
 
 
52.35
 
 
14.05
 
 
0.31
 
 
0.42
 
 
0.66
 
 
0.35
 
 
0.00
 
 
0.00
 
 
0.00
 
 
0.03
 
 
 
 
Y48a
 
 
19.11
 
 
2.90
 
 
0.96
 
 
0.03
 
 
0.23
 
 
0.04
 
 
0.00
 
 
0.31
 
 
0.08
 
 
0.39
 
 
 
 
Y48b
 
 
20.59
 
 
2.73
 
 
0.96
 
 
0.03
 
 
0.29
 
 
0.04
 
 
0.00
 
 
0.27
 
 
0.09
 
 
0.38
 
 
 
 
Y48c
 
 
17.62
 
 
3.12
 
 
0.91
 
 
0.03
 
 
0.18
 
 
0.04
 
 
0.00
 
 
0.39
 
 
0.08
 
 
0.42
 
 
 
 
MMa
 
 
25.43
 
 
1.50
 
 
0.35
 
 
0.20
 
 
0.13
 
 
0.49
 
 
0.00
 
 
0.65
 
 
0.29
 
 
1.44
 
 
 
 
MMb
 
 
29.62
 
 
1.46
 
 
0.32
 
 
0.11
 
 
0.12
 
 
0.24
 
 
0.00
 
 
0.73
 
 
0.18
 
 
2.36
 
 
 
 
SAFa
 
 
18.20
 
 
0.90
 
 
0.37
 
 
0.12
 
 
0.11
 
 
0.22
 
 
0.00
 
 
0.74
 
 
0.76
 
 
1.23
 
 
 
 
SAFb
 
 
17.85
 
 
0.94
 
 
0.43
 
 
0.21
 
 
0.11
 
 
0.64
 
 
0.00
 
 
0.80
 
 
0.57
 
 
1.83
 
 
 
 
SAFc
 
 
15.74
 
 
0.63
 
 
0.52
 
 
0.19
 
 
0.12
 
 
0.44
 
 
0.00
 
 
0.89
 
 
0.75
 
 
1.63
 
 
 
 
NAGa
 
 
11.86
 
 
1.07
 
 
0.55
 
 
0.29
 
 
0.12
 
 
0.71
 
 
0.00
 
 
0.59
 
 
0.34
 
 
1.14
 
 
 
 
NAGb
 
 
25.08
 
 
2.93
 
 
0.36
 
 
0.10
 
 
0.07
 
 
0.39
 
 
0.00
 
 
0.72
 
 
0.21
 
 
1.26
 
 
 
 
ONAa
 
 
21.16
 
 
1.20
 
 
0.40
 
 
0.14
 
 
0.12
 
 
0.46
 
 
0.02
 
 
0.71
 
 
0.49
 
 
2.65
 
 
 
 
ONAb
 
 
17.58
 
 
1.43
 
 
0.57
 
 
0.28
 
 
0.11
 
 
0.69
 
 
0.07
 
 
0.70
 
 
0.42
 
 
2.30
 
 
 
 
ONAc
 
 
18.75
 
 
1.20
 
 
0.55
 
 
0.23
 
 
0.14
 
 
0.43
 
 
0.06
 
 
0.83
 
 
0.56
 
 
2.50
 
 
 
 
HTa
 
 
24.03
 
 
1.55
 
 
0.32
 
 
0.15
 
 
0.14
 
 
0.35
 
 
0.00
 
 
0.73
 
 
0.54
 
 
1.14
 
 
 
 
HTb
 
 
23.04
 
 
1.19
 
 
0.41
 
 
0.21
 
 
0.14
 
 
0.53
 
 
0.00
 
 
0.66
 
 
0.44
 
 
1.11
 
 
 
 
HTc
 
 
20.04
 
 
0.87
 
 
0.41
 
 
0.20
 
 
0.14
 
 
0.42
 
 
0.00
 
 
0.81
 
 
0.81
 
 
1.31
 
 
 
 
 We may wish to assess the similarity between samples across
conditions. A pairwise scatter plot is produced (figure 5) to show how
replicates and samples from different biological conditions are similar
or different (using a log scale). Moreover, as the Pearson correlation
has been shown not to be relevant to measure the similarity between
replicates, the SERE statistic has been proposed as a similarity index
between RNA-Seq samples  [5] . It measures
whether the variability between samples is random Poisson variability or
higher. Pairwise SERE values are printed in the lower triangle of the
pairwise scatter plot. The value of the SERE statistic is: 
 
  0 when samples are identical (no variability at all: this may
happen in the case of a sample duplication);  
  1 for technical replicates (technical variability follows a
Poisson distribution);  
  greater than 1 for biological replicates and samples from
different biological conditions (biological variability is higher than
technical one, data are over-dispersed with respect to Poisson). The
higher the SERE value, the lower the similarity. It is expected to be
lower between biological replicates than between samples of different
biological conditions. Hence, the SERE statistic can be used to detect
inversions between samples.  
 
 
 
 
 Figure 5: Pairwise comparison of samples (not
produced when more than 12 samples). 
 
 
 
 
  3  Variability within the
experiment: data exploration 
 The main variability within the experiment is expected to come from
biological differences between the samples. This can be checked in two
ways. The first one is to perform a hierarchical clustering of the whole
sample set. This is performed after a transformation of the count data
which can be either a Variance Stabilizing Transformation (VST) or a
regularized log transformation (rlog)  [3,4] . 
 A VST is a transformation of the data that makes them homoscedastic,
meaning that the variance is then independent of the mean. It is
performed in two steps: (i) a mean-variance relationship is estimated
from the data with the same function that is used to normalize count
data and (ii) from this relationship, a transformation of the data is
performed in order to get a dataset in which the variance is independent
of the mean. The homoscedasticity is a prerequisite for the use of some
data analysis methods, such as hierarchical clustering or Principal
Component Analysis (PCA). The regularized log transformation is based on
a GLM (Generalized Linear Model) on the counts and has the same goal as
a VST but is more robust in the case when the size factors vary
widely. 
 Figure 6 shows the dendrogram obtained from VST-transformed data. An
euclidean distance is computed between samples, and the dendrogram is
built upon the Ward criterion. We expect this dendrogram to group
replicates and separate biological conditions. 
 
 
 
 Figure 6: Sample clustering based on normalized
data. 
 
 
 Another way of visualizing the experiment variability is to look at
the first principal components of the PCA, as shown on the figure 7. On
this figure, the first principal component (PC1) is expected to separate
samples from the different biological conditions, meaning that the
biological variability is the main source of variance in the data. 
 
 
 
 Figure 7: First two components of a Principal
Component Analysis, with percentages of variance associated with each
axis. 
 
 
 
 
  4  Normalization 
 Normalization aims at correcting systematic technical biases in the
data, in order to make read counts comparable across samples. The
normalization proposed by DESeq2 relies on the hypothesis that most
features are not differentially expressed. It computes a scaling factor
for each sample. Normalized read counts are obtained by dividing raw
read counts by the scaling factor associated with the sample they belong
to. Scaling factors around 1 mean (almost) no normalization is
performed. Scaling factors lower than 1 will produce normalized counts
higher than raw ones, and the other way around. Two options are
available to compute scaling factors: locfunc=“median” (default) or
locfunc=“shorth”. Here, the normalization was performed with
locfunc=“median”. 
 
 
Table 5: Normalization factors.
 
 
 
 
Size factor
 
 
2.74
 
 
2.38
 
 
1.79
 
 
1.2
 
 
0.96
 
 
1.3
 
 
1.2
 
 
0.62
 
 
1.64
 
 
1.73
 
 
1.82
 
 
0.4
 
 
0.63
 
 
0.42
 
 
0.45
 
 
1.43
 
 
1.1
 
 
1.9
 
 
1.78
 
 
2.14
 
 
0.9
 
 
1.26
 
 
1.86
 
 
1.61
 
 
0.6
 
 
0.83
 
 
0.66
 
 
0.69
 
 
0.82
 
 
0.7
 
 
0.83
 
 
0.56
 
 
0.63
 
 
0.65
 
 
0.42
 
 
0.63
 
 
0.72
 
 
 
 
 The histograms (figure 8) can help to validate the choice of the
normalization parameter (“median” or “shorth”). Under the hypothesis
that most features are not differentially expressed, each size factor
represented by a red line is expected to be close to the mode of the
distribution of the counts divided by their geometric means across
samples. 
 
 
 
 Figure 8: Diagnostic of the estimation of the size
factors. 
 
 
 The figure 9 shows that the scaling factors of DESeq2 and the total
count normalization factors may not perform similarly. 
 
 
 
 Figure 9: Plot of the estimated size factors and the
total number of reads per sample. 
 
 
 Boxplots are often used as a qualitative measure of the quality of
the normalization process, as they show how distributions are globally
affected during this process. We expect normalization to stabilize
distributions across samples. Figure 10 shows boxplots of raw (left) and
normalized (right) data respectively. 
 
 
 
 Figure 10: Boxplots of raw (left) and normalized
(right) read counts. 
 
 
 
 
  5  Differential
analysis 
 
  5.1  Modelisation 
 DESeq2 aims at fitting one linear model per feature. For this
project, the design used is counts ~ Condition and the goal is to
estimate the models’ coefficients which can be interpreted as  \(\log_2(\texttt{FC})\) . These coefficients
will then be tested to get p-values and adjusted p-values. 
 
 
  5.2  Outlier
detection 
 Model outliers are features for which at least one sample seems
unrelated to the experimental or study design. For every feature and for
every sample, the Cook’s distance  [6] 
reflects how the sample matches the model. A large value of the Cook’s
distance indicates an outlier count and p-values are not computed for
the corresponding feature. 
 
 
  5.3  Dispersions
estimation 
 The DESeq2 model assumes that the count data follow a negative
binomial distribution which is a robust alternative to the Poisson law
when data are over-dispersed (the variance is higher than the mean). The
first step of the statistical procedure is to estimate the dispersion of
the data. Its purpose is to determine the shape of the mean-variance
relationship. The default is to apply a GLM (Generalized Linear Model)
based method (fitType=“parametric”), which can handle complex designs
but may not converge in some cases. The alternative is to use
fitType=“local” as described in the original paper  [3]  or fitType=“mean”. The parameter used for
this project is fitType=“parametric”. Then, DESeq2 imposes a Cox
Reid-adjusted profile likelihood maximization  [7
and McCarthy, 2012]  and uses the maximum  a posteriori 
(MAP) of the dispersion [Wu, 2013]. 
 
 
 
 Figure 11: Dispersion estimates (left) and diagnostic
of log-normality (right). 
 
 
 The left panel on figure 11 shows the result of the dispersion
estimation step. The x- and y-axes represent the mean count value and
the estimated dispersion respectively. Black dots represent empirical
dispersion estimates for each feature (from the observed counts). The
red dots show the mean-variance relationship function (fitted dispersion
value) as estimated by the model. The blue dots are the final estimates
from the maximum  a posteriori  and are used to perform the
statistical test. Blue circles (if any) point out dispersion outliers.
These are features with a very high empirical variance (computed from
observed counts). These high dispersion values fall far from the model
estimation. For these features, the statistical test is based on the
empirical variance in order to be more conservative than with the MAP
dispersion. These features will have low chance to be declared
significant. The figure on the right panel allows to check the
hypothesis of log-normality of the dispersions. 
 
 
  5.4  Statistical test for
differential expression 
 Once the dispersion estimation and the model fitting have been done,
DESeq2 can perform the statistical testing. Figure 12 shows the
distributions of raw p-values computed by the statistical test for the
comparison(s) done. This distribution is expected to be a mixture of a
uniform distribution on  \([0,1]\)  and a
peak around 0 corresponding to the differentially expressed
features. 
 
 
 
 Figure 12: Distribution(s) of raw p-values. 
 
 
 
 
  5.5  Independent
filtering 
 DESeq2 can perform an independent filtering to increase the detection
power of differentially expressed features at the same experiment-wide
type I error. Since features with very low counts are not likely to see
significant differences typically due to high dispersion, it defines a
threshold on the mean of the normalized counts irrespective of the
biological condition. This procedure is independent because the
information about the variables in the design formula is not used  [4] . 
Table 6 reports the thresholds used for each comparison and the number
of features discarded by the independent filtering. Adjusted p-values of
discarded features are then set to NA.
 
 
Table 6: Number of features discarded by the independent filtering for
each comparison.
 
 
 
 
Test vs Ref
 
 
BaseMean Threshold
 
 
# discarded
 
 
 
 
 
 
MP30 vs MP24
 
 
0.02
 
 
203
 
 
 
 
MP36 vs MP24
 
 
0.02
 
 
203
 
 
 
 
MP48 vs MP24
 
 
0.02
 
 
203
 
 
 
 
MP72 vs MP24
 
 
0.02
 
 
203
 
 
 
 
Ycongo24 vs MP24
 
 
7.38
 
 
629
 
 
 
 
Y24 vs MP24
 
 
7.38
 
 
629
 
 
 
 
Y48 vs MP24
 
 
0.02
 
 
203
 
 
 
 
MM vs MP24
 
 
0.02
 
 
203
 
 
 
 
SAF vs MP24
 
 
0.02
 
 
203
 
 
 
 
NAG vs MP24
 
 
0.02
 
 
203
 
 
 
 
ONA vs MP24
 
 
0.02
 
 
203
 
 
 
 
HT vs MP24
 
 
0.02
 
 
203
 
 
 
 
MP36 vs MP30
 
 
4.64
 
 
487
 
 
 
 
MP48 vs MP30
 
 
0.02
 
 
203
 
 
 
 
MP72 vs MP30
 
 
0.02
 
 
203
 
 
 
 
Ycongo24 vs MP30
 
 
4.64
 
 
487
 
 
 
 
Y24 vs MP30
 
 
10.57
 
 
771
 
 
 
 
Y48 vs MP30
 
 
0.02
 
 
203
 
 
 
 
MM vs MP30
 
 
0.02
 
 
203
 
 
 
 
SAF vs MP30
 
 
0.02
 
 
203
 
 
 
 
NAG vs MP30
 
 
0.02
 
 
203
 
 
 
 
ONA vs MP30
 
 
0.02
 
 
203
 
 
 
 
HT vs MP30
 
 
0.02
 
 
203
 
 
 
 
MP48 vs MP36
 
 
2.42
 
 
345
 
 
 
 
MP72 vs MP36
 
 
0.02
 
 
203
 
 
 
 
Ycongo24 vs MP36
 
 
4.64
 
 
487
 
 
 
 
Y24 vs MP36
 
 
0.02
 
 
203
 
 
 
 
Y48 vs MP36
 
 
0.02
 
 
203
 
 
 
 
MM vs MP36
 
 
0.02
 
 
203
 
 
 
 
SAF vs MP36
 
 
0.02
 
 
203
 
 
 
 
NAG vs MP36
 
 
0.02
 
 
203
 
 
 
 
ONA vs MP36
 
 
0.02
 
 
203
 
 
 
 
HT vs MP36
 
 
0.02
 
 
203
 
 
 
 
MP72 vs MP48
 
 
0.02
 
 
203
 
 
 
 
Ycongo24 vs MP48
 
 
0.02
 
 
203
 
 
 
 
Y24 vs MP48
 
 
0.02
 
 
203
 
 
 
 
Y48 vs MP48
 
 
0.02
 
 
203
 
 
 
 
MM vs MP48
 
 
0.02
 
 
203
 
 
 
 
SAF vs MP48
 
 
0.02
 
 
203
 
 
 
 
NAG vs MP48
 
 
0.02
 
 
203
 
 
 
 
ONA vs MP48
 
 
0.02
 
 
203
 
 
 
 
HT vs MP48
 
 
0.02
 
 
203
 
 
 
 
Ycongo24 vs MP72
 
 
0.02
 
 
203
 
 
 
 
Y24 vs MP72
 
 
0.02
 
 
203
 
 
 
 
Y48 vs MP72
 
 
0.02
 
 
203
 
 
 
 
MM vs MP72
 
 
0.02
 
 
203
 
 
 
 
SAF vs MP72
 
 
0.02
 
 
203
 
 
 
 
NAG vs MP72
 
 
0.02
 
 
203
 
 
 
 
ONA vs MP72
 
 
0.02
 
 
203
 
 
 
 
HT vs MP72
 
 
0.02
 
 
203
 
 
 
 
Y24 vs Ycongo24
 
 
0.02
 
 
203
 
 
 
 
Y48 vs Ycongo24
 
 
0.02
 
 
203
 
 
 
 
MM vs Ycongo24
 
 
0.02
 
 
203
 
 
 
 
SAF vs Ycongo24
 
 
0.02
 
 
203
 
 
 
 
NAG vs Ycongo24
 
 
0.02
 
 
203
 
 
 
 
ONA vs Ycongo24
 
 
0.02
 
 
203
 
 
 
 
HT vs Ycongo24
 
 
0.02
 
 
203
 
 
 
 
Y48 vs Y24
 
 
0.02
 
 
203
 
 
 
 
MM vs Y24
 
 
0.02
 
 
203
 
 
 
 
SAF vs Y24
 
 
0.02
 
 
203
 
 
 
 
NAG vs Y24
 
 
0.02
 
 
203
 
 
 
 
ONA vs Y24
 
 
0.02
 
 
203
 
 
 
 
HT vs Y24
 
 
0.02
 
 
203
 
 
 
 
MM vs Y48
 
 
0.02
 
 
203
 
 
 
 
SAF vs Y48
 
 
0.02
 
 
203
 
 
 
 
NAG vs Y48
 
 
0.02
 
 
203
 
 
 
 
ONA vs Y48
 
 
0.02
 
 
203
 
 
 
 
HT vs Y48
 
 
0.02
 
 
203
 
 
 
 
SAF vs MM
 
 
0.02
 
 
203
 
 
 
 
NAG vs MM
 
 
4.64
 
 
487
 
 
 
 
ONA vs MM
 
 
0.02
 
 
203
 
 
 
 
HT vs MM
 
 
0.02
 
 
203
 
 
 
 
NAG vs SAF
 
 
0.02
 
 
203
 
 
 
 
ONA vs SAF
 
 
0.02
 
 
203
 
 
 
 
HT vs SAF
 
 
0.02
 
 
203
 
 
 
 
ONA vs NAG
 
 
0.02
 
 
203
 
 
 
 
HT vs NAG
 
 
0.02
 
 
203
 
 
 
 
HT vs ONA
 
 
0.02
 
 
203
 
 
 
 
 
 
  5.6  Final results 
 A p-value adjustment is performed to take into account multiple
testing and control the false positive rate to a chosen level  \(\alpha\) . For this analysis, a BH p-value
adjustment was performed  [8 and BY2001] 
and the level of controlled false positive rate was set to 0.05. 
 
 
Table 7: Number of up-, down- and total number of differentially
expressed features for each comparison.
 
 
 
 
Test vs Ref
 
 
# down
 
 
# up
 
 
# total
 
 
 
 
 
 
MP30 vs MP24
 
 
692
 
 
998
 
 
1690
 
 
 
 
MP36 vs MP24
 
 
1624
 
 
1604
 
 
3228
 
 
 
 
MP48 vs MP24
 
 
2061
 
 
2278
 
 
4339
 
 
 
 
MP72 vs MP24
 
 
2406
 
 
2691
 
 
5097
 
 
 
 
Ycongo24 vs MP24
 
 
386
 
 
412
 
 
798
 
 
 
 
Y24 vs MP24
 
 
442
 
 
581
 
 
1023
 
 
 
 
Y48 vs MP24
 
 
2330
 
 
2695
 
 
5025
 
 
 
 
MM vs MP24
 
 
2182
 
 
2336
 
 
4518
 
 
 
 
SAF vs MP24
 
 
2361
 
 
2522
 
 
4883
 
 
 
 
NAG vs MP24
 
 
1837
 
 
2156
 
 
3993
 
 
 
 
ONA vs MP24
 
 
2195
 
 
2444
 
 
4639
 
 
 
 
HT vs MP24
 
 
2354
 
 
2733
 
 
5087
 
 
 
 
MP36 vs MP30
 
 
849
 
 
865
 
 
1714
 
 
 
 
MP48 vs MP30
 
 
1817
 
 
1988
 
 
3805
 
 
 
 
MP72 vs MP30
 
 
2332
 
 
2351
 
 
4683
 
 
 
 
Ycongo24 vs MP30
 
 
888
 
 
752
 
 
1640
 
 
 
 
Y24 vs MP30
 
 
790
 
 
697
 
 
1487
 
 
 
 
Y48 vs MP30
 
 
2217
 
 
2268
 
 
4485
 
 
 
 
MM vs MP30
 
 
2202
 
 
2225
 
 
4427
 
 
 
 
SAF vs MP30
 
 
2239
 
 
2264
 
 
4503
 
 
 
 
NAG vs MP30
 
 
1916
 
 
1998
 
 
3914
 
 
 
 
ONA vs MP30
 
 
2195
 
 
2316
 
 
4511
 
 
 
 
HT vs MP30
 
 
2332
 
 
2573
 
 
4905
 
 
 
 
MP48 vs MP36
 
 
713
 
 
1126
 
 
1839
 
 
 
 
MP72 vs MP36
 
 
1866
 
 
1946
 
 
3812
 
 
 
 
Ycongo24 vs MP36
 
 
1500
 
 
1729
 
 
3229
 
 
 
 
Y24 vs MP36
 
 
1417
 
 
1837
 
 
3254
 
 
 
 
Y48 vs MP36
 
 
1873
 
 
1934
 
 
3807
 
 
 
 
MM vs MP36
 
 
1934
 
 
2099
 
 
4033
 
 
 
 
SAF vs MP36
 
 
1879
 
 
2017
 
 
3896
 
 
 
 
NAG vs MP36
 
 
1616
 
 
1888
 
 
3504
 
 
 
 
ONA vs MP36
 
 
1915
 
 
2185
 
 
4100
 
 
 
 
HT vs MP36
 
 
2038
 
 
2519
 
 
4557
 
 
 
 
MP72 vs MP48
 
 
2099
 
 
1811
 
 
3910
 
 
 
 
Ycongo24 vs MP48
 
 
2286
 
 
2161
 
 
4447
 
 
 
 
Y24 vs MP48
 
 
2225
 
 
2246
 
 
4471
 
 
 
 
Y48 vs MP48
 
 
1849
 
 
1675
 
 
3524
 
 
 
 
MM vs MP48
 
 
1980
 
 
1903
 
 
3883
 
 
 
 
SAF vs MP48
 
 
2053
 
 
1798
 
 
3851
 
 
 
 
NAG vs MP48
 
 
1956
 
 
1860
 
 
3816
 
 
 
 
ONA vs MP48
 
 
2135
 
 
2069
 
 
4204
 
 
 
 
HT vs MP48
 
 
2140
 
 
2333
 
 
4473
 
 
 
 
Ycongo24 vs MP72
 
 
2581
 
 
2393
 
 
4974
 
 
 
 
Y24 vs MP72
 
 
2542
 
 
2478
 
 
5020
 
 
 
 
Y48 vs MP72
 
 
1919
 
 
1990
 
 
3909
 
 
 
 
MM vs MP72
 
 
1865
 
 
1875
 
 
3740
 
 
 
 
SAF vs MP72
 
 
1699
 
 
1612
 
 
3311
 
 
 
 
NAG vs MP72
 
 
1814
 
 
1887
 
 
3701
 
 
 
 
ONA vs MP72
 
 
1843
 
 
1835
 
 
3678
 
 
 
 
HT vs MP72
 
 
1851
 
 
2173
 
 
4024
 
 
 
 
Y24 vs Ycongo24
 
 
5
 
 
1
 
 
6
 
 
 
 
Y48 vs Ycongo24
 
 
2317
 
 
2542
 
 
4859
 
 
 
 
MM vs Ycongo24
 
 
2264
 
 
2353
 
 
4617
 
 
 
 
SAF vs Ycongo24
 
 
2384
 
 
2534
 
 
4918
 
 
 
 
NAG vs Ycongo24
 
 
1956
 
 
2180
 
 
4136
 
 
 
 
ONA vs Ycongo24
 
 
2305
 
 
2505
 
 
4810
 
 
 
 
HT vs Ycongo24
 
 
2437
 
 
2752
 
 
5189
 
 
 
 
Y48 vs Y24
 
 
2404
 
 
2541
 
 
4945
 
 
 
 
MM vs Y24
 
 
2333
 
 
2313
 
 
4646
 
 
 
 
SAF vs Y24
 
 
2471
 
 
2484
 
 
4955
 
 
 
 
NAG vs Y24
 
 
2072
 
 
2117
 
 
4189
 
 
 
 
ONA vs Y24
 
 
2378
 
 
2466
 
 
4844
 
 
 
 
HT vs Y24
 
 
2483
 
 
2732
 
 
5215
 
 
 
 
MM vs Y48
 
 
2357
 
 
2318
 
 
4675
 
 
 
 
SAF vs Y48
 
 
2295
 
 
2283
 
 
4578
 
 
 
 
NAG vs Y48
 
 
2288
 
 
2228
 
 
4516
 
 
 
 
ONA vs Y48
 
 
2317
 
 
2371
 
 
4688
 
 
 
 
HT vs Y48
 
 
2240
 
 
2560
 
 
4800
 
 
 
 
SAF vs MM
 
 
1027
 
 
1027
 
 
2054
 
 
 
 
NAG vs MM
 
 
838
 
 
731
 
 
1569
 
 
 
 
ONA vs MM
 
 
1029
 
 
1046
 
 
2075
 
 
 
 
HT vs MM
 
 
889
 
 
1218
 
 
2107
 
 
 
 
NAG vs SAF
 
 
1093
 
 
1179
 
 
2272
 
 
 
 
ONA vs SAF
 
 
537
 
 
543
 
 
1080
 
 
 
 
HT vs SAF
 
 
639
 
 
1121
 
 
1760
 
 
 
 
ONA vs NAG
 
 
1071
 
 
1057
 
 
2128
 
 
 
 
HT vs NAG
 
 
1390
 
 
1600
 
 
2990
 
 
 
 
HT vs ONA
 
 
528
 
 
1037
 
 
1565
 
 
 
 
 Figure 13 represents the MA-plot of the data for the comparisons
done, where differentially expressed features are highlighted in red. A
MA-plot represents the log ratio of differential expression as a
function of the mean intensity for each feature. Triangles correspond to
features having a too low/high  \(\log_2(\text{FC})\)  to be displayed on the
plot. 
 
 
 
 Figure 13: MA-plot(s) of each comparison. Red dots
represent significantly differentially expressed features. 
 
 
 Figure 14 shows the volcano plots for the comparisons performed and
differentially expressed features are still highlighted in red. A
volcano plot represents the log of the adjusted P value as a function of
the log ratio of differential expression. 
 
 
 
 Figure 14: Volcano plot(s) of each comparison. Red
dots represent significantly differentially expressed features. 
 
 
 Note that the log2(Fold-Changes) are shrunk using the “ashr” method
that has been shown to be more robust than the original “normal” method
 [9] . 
 Full results as well as lists of differentially expressed features
are provided in the following text files which can be easily read in a
spreadsheet. For each comparison: 
 
 TestVsRef.complete.txt contains results for all the features; 
 TestVsRef.up.txt contains results for significantly up-regulated
features. Features are ordered from the most significant adjusted
p-value to the less significant one; 
 TestVsRef.down.txt contains results for significantly down-regulated
features. Features are ordered from the most significant adjusted
p-value to the less significant one. 
 
 These files contain the following columns: 
 
 Id: unique feature identifier; 
 sampleName: raw counts per sample; 
 norm.sampleName: rounded normalized counts per sample; 
 baseMean: base mean over all samples; 
 MP24, MP30, MP36, MP48, MP72, Ycongo24, Y24, Y48, MM, SAF, NAG, ONA
and HT: means (rounded) of normalized counts of the biological
conditions; 
 FoldChange: fold change of expression, calculated as  \(2^{\log_2(\text{FC})}\) ; 
 log2FoldChange:  \(\log_2(\text{FC})\)  as estimated by the GLM
model. It reflects the differential expression between Test and Ref and
can be interpreted as  \(\log_2(\frac{\text{Test}}{\text{Ref}})\) .
If this value is:
 
 around 0: the feature expression is similar in both conditions; 
 positive: the feature is up-regulated ( \(\text{Test} &gt; \text{Ref}\) ); 
 negative: the feature is down-regulated ( \(\text{Test} &lt; \text{Ref}\) ); 
  
 stat: Wald statistic for the coefficient tested; 
 pvalue: raw p-value from the statistical test; 
 padj: adjusted p-value on which the cut-off  \(\alpha\)  is applied; 
 dispGeneEst: dispersion parameter estimated from feature counts
(i.e. black dots on figure 11); 
 dispFit: dispersion parameter estimated from the model (i.e. red
dots on figure 11); 
 dispMAP: dispersion parameter estimated from the Maximum  A
Posteriori  model; 
 dispersion: final dispersion parameter used to perform the test
(i.e. blue dots and circles on figure 11); 
 betaConv: convergence of the coefficients of the model (TRUE or
FALSE); 
 maxCooks: maximum Cook’s distance of the feature. 
 
 
 
 
  6  R session information
and parameters 
 The versions of the R software and Bioconductor packages used for
this analysis are listed below. It is important to save them if one
wants to re-perform the analysis in the same conditions. 
 
 R version 4.2.2 (2022-10-31 ucrt), x86_64-w64-mingw32 
 
 Locale: LC_COLLATE=French_France.utf8, LC_CTYPE=French_France.utf8,
LC_MONETARY=French_France.utf8, LC_NUMERIC=C,
LC_TIME=French_France.utf8 
 
 Running under: Windows 10 x64 (build 19045) 
 
 Matrix products: default 
 
 Base packages: base, datasets, graphics, grDevices, methods, stats,
stats4, utils 
 
 Other packages: ashr 2.2-54, Biobase 2.56.0, BiocGenerics 0.42.0,
DESeq2 1.36.0, edgeR 3.40.1, GenomeInfoDb 1.32.4, GenomicRanges 1.48.0,
ggplot2 3.4.1, IRanges 2.30.1, kableExtra 1.3.4, limma 3.54.0,
MatrixGenerics 1.8.1, matrixStats 0.63.0, S4Vectors 0.34.0, SARTools
1.8.1, SummarizedExperiment 1.26.1 
 
 Loaded via a namespace (and not attached): annotate 1.74.0,
AnnotationDbi 1.58.0, BiocManager 1.30.19, BiocParallel 1.30.4,
Biostrings 2.64.1, bit 4.0.5, bit64 4.0.5, bitops 1.0-7, blob 1.2.3,
bslib 0.4.2, cachem 1.0.7, callr 3.7.3, cli 3.6.0, codetools 0.2-19,
colorspace 2.1-0, compiler 4.2.2, crayon 1.5.2, DBI 1.1.3, DelayedArray
0.22.0, devtools 2.4.5, digest 0.6.31, dplyr 1.1.0, ellipsis 0.3.2,
evaluate 0.20, fansi 1.0.4, farver 2.1.1, fastmap 1.1.1, fs 1.6.1,
genefilter 1.78.0, geneplotter 1.74.0, generics 0.1.3, GenomeInfoDbData
1.2.8, GGally 2.1.2, ggdendro 0.1.23, ggrepel 0.9.3, glue 1.6.2, grid
4.2.2, gridExtra 2.3, gtable 0.3.1, highr 0.10, htmltools 0.5.4,
htmlwidgets 1.6.1, httpuv 1.6.9, httr 1.4.5, invgamma 1.1, irlba
2.3.5.1, jquerylib 0.1.4, jsonlite 1.8.4, KEGGREST 1.36.3, knitr 1.42,
labeling 0.4.2, later 1.3.0, lattice 0.20-45, lifecycle 1.0.3, locfit
1.5-9.7, magrittr 2.0.3, MASS 7.3-58.2, Matrix 1.5-3, memoise 2.0.1,
mime 0.12, miniUI 0.1.1.1, mixsqp 0.3-48, munsell 0.5.0, parallel 4.2.2,
pillar 1.8.1, pkgbuild 1.4.0, pkgconfig 2.0.3, pkgload 1.3.2, plyr
1.8.8, png 0.1-8, prettyunits 1.1.1, processx 3.8.0, profvis 0.3.7,
promises 1.2.0.1, ps 1.7.2, purrr 1.0.1, R6 2.5.1, RColorBrewer 1.1-3,
Rcpp 1.0.10, RCurl 1.98-1.10, remotes 2.4.2, reshape 0.8.9, rlang 1.0.6,
rmarkdown 2.20, RSQLite 2.3.0, rstudioapi 0.14, rvest 1.0.3, sass 0.4.5,
scales 1.2.1, sessioninfo 1.2.2, shiny 1.7.4, splines 4.2.2, SQUAREM
2021.1, stringi 1.7.12, stringr 1.5.0, survival 3.5-3, svglite 2.1.1,
systemfonts 1.0.4, tibble 3.2.0, tidyselect 1.2.0, tools 4.2.2,
truncnorm 1.0-8, urlchecker 1.0.1, usethis 2.1.6, utf8 1.2.3, vctrs
0.5.2, viridisLite 0.4.1, webshot 0.5.4, withr 2.5.0, xfun 0.37, XML
3.99-0.13, xml2 1.3.3, xtable 1.8-4, XVector 0.36.0, yaml 2.3.7,
zlibbioc 1.42.0 
 
 Parameter values used for this analysis are: 
 
 workDir: ./ 
 projectName: Samy phage OSMAC-RNAseq 
 author: Stéphanie Bury-Moné 
 targetFile: target_SAM.txt 
 rawDir: ./Counts/ 
 featuresToRemove: alignment_not_unique, ambiguous, no_feature,
not_aligned, too_low_aQual 
 varInt: Condition 
 condRef: MP24 
 batch: NULL 
 fitType: parametric 
 cooksCutoff: TRUE 
 independentFiltering: TRUE 
 alpha: 0.05 
 pAdjustMethod: BH 
 typeTrans: VST 
 locfunc: median 
 colors: #f3c300, #875692, #f38400, #a1caf1, #be0032, #c2b280,
#848482, #008856, #e68fac, #0067a5, #f99379, #604e97, lightsalmon3 
 
 
 
 Bibliography 
 
 
 1.   R
Core Team.   R: A language and
environment for statistical computing  . Vienna, Austria : R
Foundation for Statistical Computing, 2017 : 
 
 
 2.   Gentleman RC, Carey VJ, Bates DM,  et
al.   Bioconductor: Open
software development for computational biology and bioinformatics .
 Genome Biology  2004 ; 5 : R80. 
 
 
 3.   Anders S, Huber W.  Differential
expression analysis for sequence count data .  Genome Biology 
2010 ; 11 : R106. 
 
 
 4.   Love
MI, Huber W, Anders S.  Moderated estimation of
fold change and dispersion for RNA-seq data with DESeq2 .  Genome
Biology  2014 ; 15 : 550. 
 
 
 5.   Schulze SK, Kanwar R, Gölzenleuchter M,  et
al.   SERE:
Single-parameter quality control and sample comparison for RNA-seq .
 BMC Genomics  2012 ; 13 : 524. 
 
 
 6.   Cook
RD.  Detection of
influential observation in linear regression .  Technometrics 
1977 ; 19 : 15–18. 
 
 
 7.   Cox
DR, Reid N.  Parameter
orthogonality and approximate conditional inference .  Journal of
the Royal Statistical Society. Series B (Methodological)  1987 ; 49
: 1–39. 
 
 
 8.   Benjamini Y, Hochberg Y.  Controlling the false
discovery rate: A practical and powerful approach to multiple
testing .  Journal of the Royal Statistical Society. Series B
(Methodological)  1995 ; 57 : 289–300. 
 
 
 9.   Stephens M.   False discovery rates: a new deal  .
 Biostatistics  2016 ; 18 : 275–294. 
 
 
 


 
 

 

 

 

 

 

 

 
 

 
 
