# Supplementary figures and images for: Prophage induction can facilitate the in vitro dispersal of multicellular Streptomyces structures
Source: PLoS Biol. 2024 Jul 25;22(7):e3002725. doi: 10.1371/journal.pbio.3002725 (PMC11302927; doi:10.1371/journal.pbio.3002725)

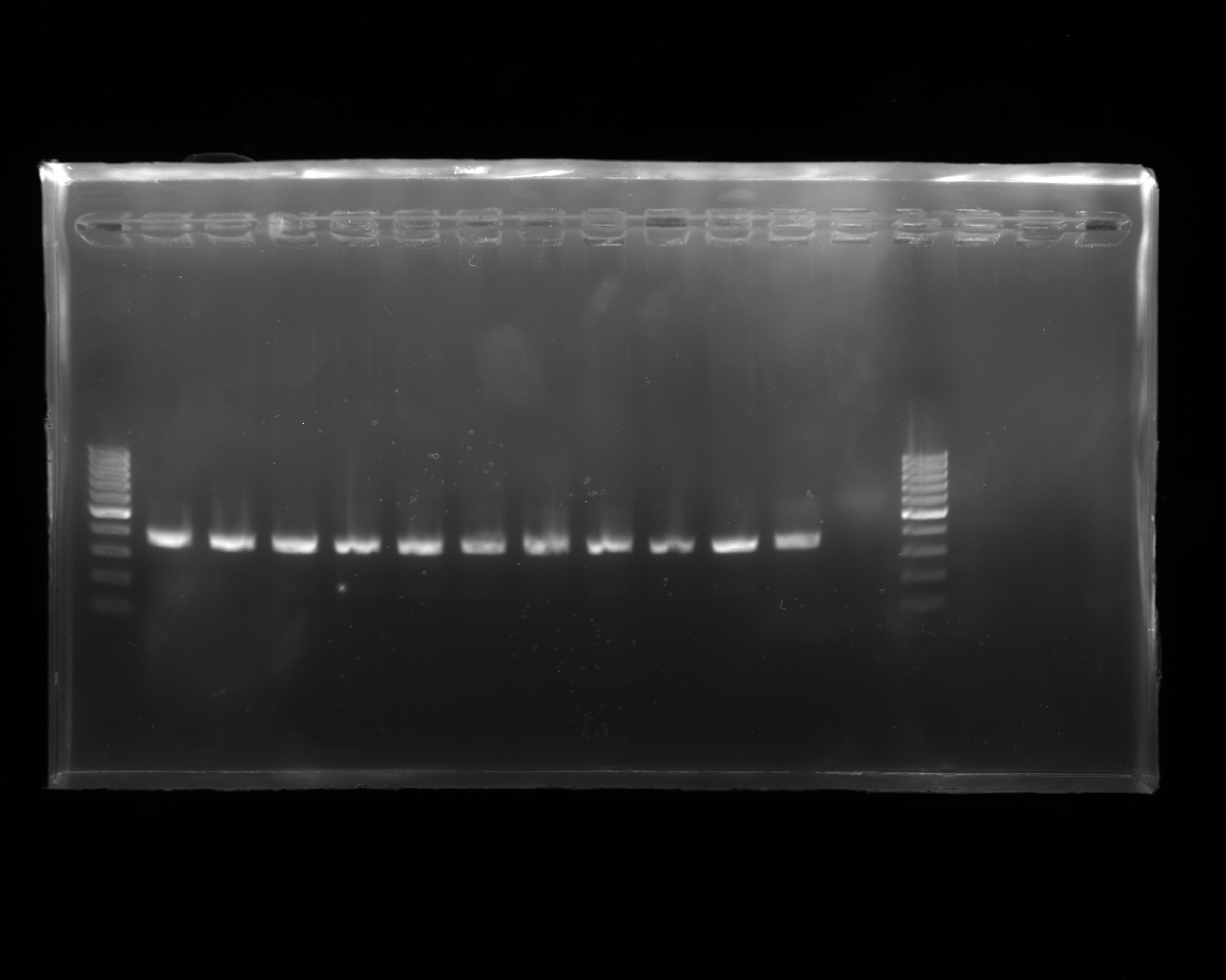

Supplement: S1 Raw Image — (JPG) [file pbio.3002725.s021.jpg]
