# Supplementary material for: Prophage induction can facilitate the in vitro dispersal of multicellular Streptomyces structures
Source: PLoS Biol. 2024 Jul 25;22(7):e3002725. doi: 10.1371/journal.pbio.3002725 (PMC11302927; doi:10.1371/journal.pbio.3002725)
Supplement: Attachment — Submitted filename: Response_to_Referees_PBIOLOGY-D-24-00044R1.pdf [file pbio.3002725.s022.pdf]

PLOS Biology

Prophage induction can facilitate the in vitro dispersal of multicellular bacteria

Ref: PBIOLOGY-D-24-00044R1
Authors: Jaffal *et al.*
Revision date: 2024/05/15
Corresponding author: Stéphanie Bury-Moné

Editorial requests

In light of the reviews, which you will find at the end of this email, we would like to invite you to revise the work to thoroughly address the reviewers' reports. As you will see below, although the reviewers see the potential interest in your work, there are still concerns that should be addressed. Overall, there is sufficient interest for us to consider the study further, but some revision will be necessary for publication in PLOS Biology. Reviewer #1 suggests that the taxonomy and characteristics of the prophage should be expanded, and reviewers #2 and #3 would like to see more in depth mechanistic insights, such as the mechanism of induction. Additionally, during cross-comments (here paraphrased), reviewers suggested to evaluate if NAG can be an inducible signal. If verified, this finding could be linked to programmed cell death. Additional suggestions include fluorescence in situ hybridisation or live/dead staining to evaluate if prophage induction leads to lysis of fragments in the mycelium. You should also consider previous reports of prophages in actinobacteria such as doi: 10.1016/j.xgen.2022.100213 and doi: 10.1038/s41598-023-30829-z. IMPORTANT: as this is submitted as a Short Report, we do not require you to decipher fully the mechanism of induction. HOWEVER, for publication in PLOS Biology, we do require additional experimental and phylogenetic analysis to better characterise this phage and its induction, following the recommendations of the reviewers.

Our response: We thank the editor and referees for their favorable evaluation of our work, and for their valuable suggestions. We have addressed the main requests, including: i) conducting a comprehensive analysis of virus classification, ii) incorporating experiments to assess NAG induction and enhance understanding of viral replication within the colony, and iii) ensuring completion of the bibliography.

Point-by-point reply to referee #1

Referee #1, Pr Dennis Claessen, stated:

Streptomyces bacteria are well-known for their production of specialized metabolites vital in medicine and agriculture, and their interactions with bacteriophages remain poorly understood. This study focused on the 'Samy' prophage in *Streptomyces ambofaciens* and revealed its simultaneous production with activation of other genetic elements and its ability to increase bacterial dispersal under stress. This research sheds new light on the role of bacteriophage infections in the dynamics of multicellular aggregates. The manuscript is well-written, and the authors bring a new element to the life cycle of these bacteria under influence of stress. The experiments have been well designed and carried out, and the major conclusions are valid based on the presented data.

Our response: We thank Pr Claessen for his positive and detailed review of our article.

And then the referee stated:

I have a few suggestions to further improve this story, which overall I am very much impressed with:
- In line 47, the authors imply that all identified *Streptomyces phages* are double stranded DNA viruses. But are really "all" *Streptomyces* phages double stranded DNA viruses? I think this sentence should be rephrases to avoid ambiguity.

Our response: To avoid this ambiguity, we have rephrased the sentence as follows:
Line 47: "The *Streptomyces* phages identified so far are double stranded DNA viruses belonging to Caudoviricetes class or *Tectiviridae* family, with the possible exception of an RNA virus detected in a *Streptomyces* transcriptome (7)".

- In the introduction, nothing is mentioned on how a bacteriophage infects a multicellular bacteria. Most research is performed on unicellular model organisms, but can the authors speculate on how phages infect a multicellular bacteria? For example, can a phage travel through the hyphae? If one part of a mycelium is infected, will the whole mycelial aggregate lyse? It would be nice to speculate a bit on this infection of multicellular bacteria in the introduction.

Our response: Driven by the referee's comment, we added two sentences in the introduction:
Line 49: "Nevertheless, the mechanisms by which bacteriophages recognize, attach to, multiply into and eventually propagate within multicellular mycelia remain largely unknown."
"Exploring how the multicellular nature and complex differentiation of *Streptomyces* can influence the viral cycle, potentially leading to partial and/or transient resistance to phages, is a promising avenue for research (9,10)."

- When talking about *Streptomyces* having a viral genetic reservoir in line 64, the authors refer to a paper that identified prophages in seven *Streptomyces* strains, including *S. coelicolor* to my understanding. However, in line 314, the authors state that *S. coelicolor* does not have a predicted complete prophage nor Samy. Could the others comment on this? Could it be that the used conditions may not activate the prophage?

Our response: PHASTER (http://phaster.ca/) analysis conducted on the *S. coelicolor* A3(2) genome prophage sequences are identified but they correspond to incomplete/defective prophages (**Figure R1**). Nevertheless, when analyzing the supplementary Table S3 from the Sharma *et al.* article (https://doi.org/10.1038/s41598-023-30829-z), we noticed that a prophage sequence was predicted on one of the *S. coelicolor* A3(2) plasmids using another software (VIBRANT). In case of doubt, we prefer not to take a position on the question of the presence or absence of complete (and possibly active) prophages in this strain, as this seems to depend very much on the type of prediction tool used. Moreover, as indicated by the referee, it cannot be ruled out that conditions we used do not allow these sequences to be expressed. To clarify this point, the initial sentence at line 64 was removed (see next point), and the sentence line 347 was rephrased as followed:
"Interestingly, the formation of cell aggregates during growth in BM medium was also observed with *S. coelicolor* A(3)2, another *Streptomyces* species that does not contain Samy prophage".

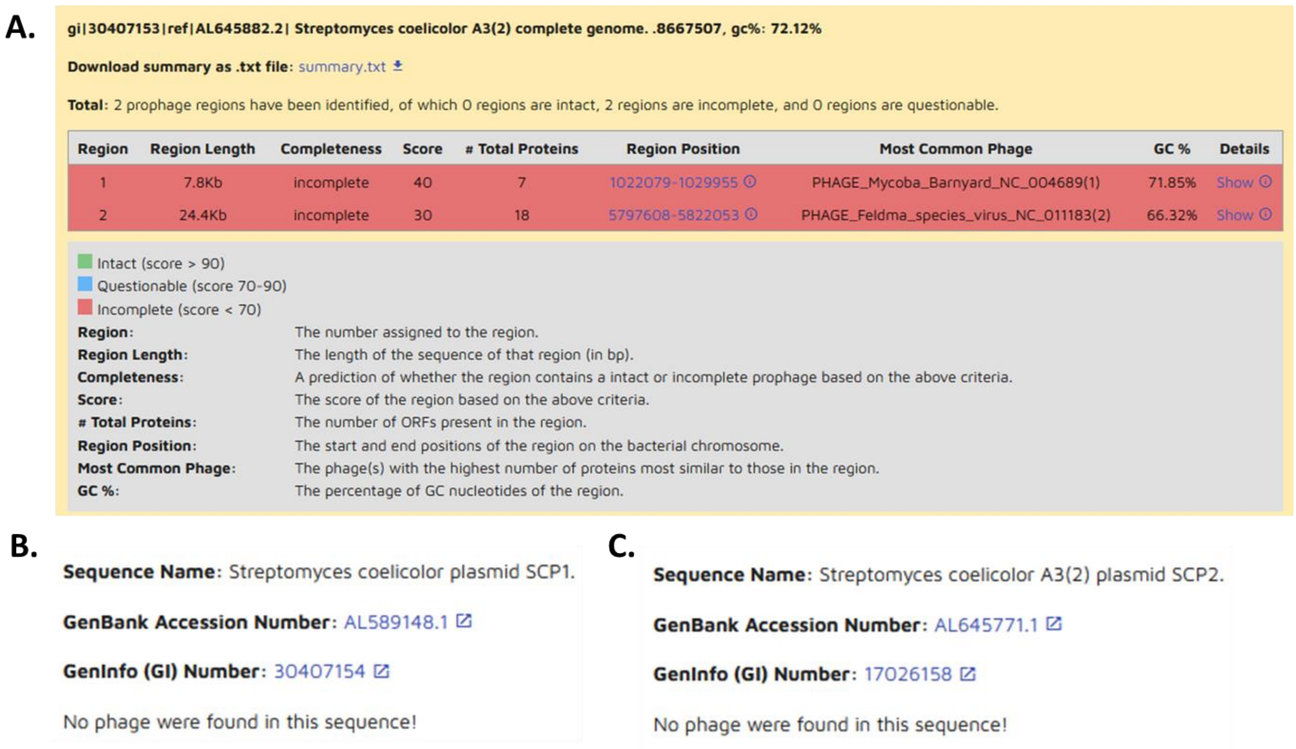

Region	Region Length	Completeness	Score	# Total Proteins	Region Position	Most Common Phage	GC %	Details
1	7.8Kb	incomplete	40	7	1022079-1029955	PHAGE_Mycoba_Barnyard_NC_004689(1)	71.85%	Show
2	24.4Kb	incomplete	30	18	5797608-5822053	PHAGE_Feldma_species_virus_NC_011183(2)	66.32%	Show

Figure R1: PHASTER predictions of prophage content in the *S. coelicolor* A3(2) chromosome (A) and plasmids (B, C)

In addition, this paper (https://doi.org/10.1016/j.xgen.2022.100213) might be a nice reference to show that almost 50% of all actinobacteria have a predicted prophage. Please make the distinction between bioinformatics suggesting a prophage and experimentally verifying the computational results.

Our response: We agree that it seems interesting to quote the global studies by Seshadri *et al.* (https://doi.org/10.1016/j.xgen.2022.100213) and Sharma *et al.* (https://doi.org/10.1038/s41598-023-30829-z). We have thus removed the initial sentence and referenced these comprehensive studies, as follows:

Line 69: "Indeed, prophage-like sequences are detected in about half of the Actinobacteria (26,27), including 62.4% of the *Streptomyces* genomes (26). While experimental validation is necessary to assess the functionality of these regions, this suggests a significant role for temperate phages in shaping the population dynamics of these bacteria."

We also notice that Sharma *et al.* detected prophage-like sequences in the pSAM1 plasmid (Supplementary Table S3 - Sheet 2 from https://doi.org/10.1038/s41598-023-30829-z). We therefore added this information in the revised version of the manuscript as followed:

Line 174: "the pSAM1 plasmid which was predicted to contain a prophage-like sequence (26)"

- In line 72, the authors say that prophages are produced, but I would suggest to change the word to "induce" under these conditions.

Our response: We agree with the referee's suggestion and have replaced "produced" with "induced".

- The supplementary figures are not in the correct order. So, Figure S2 should actually be S1 in the text etc. Please check this for the whole manuscript.

Our response: We thank the referee for pointing out this discrepancy. The figure shown in S1 was in fact S7. We have renumbered the figures in the supplementary file, and corrected the main text so that there are no more inconsistencies.

- I think the taxonomy and characteristics of Samy should be elaborated. In the material and methods from line 511-522 the authors speak of infection assays, but did you also perform plaque assays? I did not understand why these assays were grown for a week, while the common Streptomyces **plaque assays** are incubated for 1 day at 30 degrees with really pretty results. This could also explain why the authors did not see any clear infections of Samy on the tested *Streptomyces* strains. I would suggest to repeat this experiment with DNB or GYM plaque assays and perform a double agar overlay assay. Can Samy infect the *S. ambofaciens* strains without the prophage and the DSMZ strain of *S. ambofaciens*? If yes or not, please also explain in the text in line 286-292.

Our answer: Driven by these remarks, we explored Samy's classification (see next point) and carried out additional infection assays. We performed infection assays with MP Biomedicals™ Nutrient Broth (091007917) based-media which has the same composition than Difco Nutrient Broth (DNB *i.e.* Beef Extract 3 g/L, Peptone 5 g/L) and incubated the plates 1 day at 30°C. In this condition, we observed a lysis halo with *S. ambofaciens* ATCC 23877 strain as the host, but not with the other strains. These findings have been added to the article (**S5.D Fig**). These observations indicate that the infection sensitivity is significantly influenced by the experimental procedure. This may also be due to the fact that Samy is a temperate phage, which could further complicate the visibility of lysis plaques. Overall, we had to concentrate the phage by overnight centrifugation to detect plaques.

We modified the manuscript as followed:

Line 321: "Additionally, Samy virions are infectious since we observed lysis using *Streptomyces lividans* TK24 and *S. ambofaciens* ATCC 23877 strains as hosts (**S5.C&D Fig**), but not four other strains listed in the **S4 Table**. We noticed variations in Samy infection efficiency across procedures and experiments, along with the necessity to concentrate the viral particles through overnight centrifugation for plaque observation. These observations suggest either low infectivity, low burst size and/or challenges in plaque detection due to the phage's temperate nature."

Additionally, we have also constructed a conjugative vector containing the Samy integrase and its *attP* site. This non-replicating vector is effective in providing apramycin resistance in a panel of *Streptomyces* species/strains including *S. ambofaciens* DSM 406970 strain. This suggests that the integration step is not the limiting one during Samy infection. The characterization of this tool is beyond the scope of this article and will be the subject of a distinct publication.

- Next to that, is Samy classified as a new species? Since line 71 says it is a novel phage, please also explain to which genus and family this phage belongs. Since novel phages are classified between >75 and <95 genome similarity according to ICTV, also provide data to prove that this actually is a novel phage, which can be done with VIRIDIC for example. Lastly in figure 1, why is Samy compared to phiC31. This is a common and well known *Streptomyces* phage, but is this also the phage that belong in the same genus as Samy? Please compare Samy to phages in the same genus in figure 1C.

Our answer: The cluster analysis kindly performed by the "actinobacteriophage database" community indicates that Samy does not cluster with any known phages, meaning that it is a 'singleton' (https://phagesdb.org/phages/Samy/). Moreover the study by Sharma *et al.* also found that Samy prophage form a unique cluster that matches perfectly with a single actinophage (its own sequence

which was already deposited in the actinobacteriophage database at the time of their study; Supplementary Table S4 from https://doi.org/10.1038/s41598-023-30829-z).

Prompted by the referee's suggestion, we conducted an extensive analysis of Samy's classification using VIPTree and VirClust. The results are included in the revised version of the manuscript (**S7 Fig.**).

We included the following paragraph in the M&M section:

Line 608: "Samy phage classification was carried out following to the guidelines set forth by the International Committee on Taxonomy of Viruses (ICTV) (64). First, Samy nucleotide sequences underwent initial comparison with the "nucleotide collection (nt/nr)" database [Organism "Viruses (taxid:10239)"] using BLASTn (65) to identify potential related phage isolates at the species and/or genus taxonomic levels. Since only minimal similarities were identified this way (76% identity with a region of 429 bp of the *Gordiana* phage Mollymur), further investigations were conducted at the family rank using VIPTree (66) and VirClust (42), two predicted proteome-based clustering tools. A hierarchical clustering between Samy and all the reference viral sequences of the GenomeNet/Virus-Host DB (67) was first calculated based on tBLASTx comparisons on their whole proteomes using VIPTree (https://www.genome.jp/viptree/; version 4.0, default parameters). All the viral sequences of the VIPTree cluster including Samy (*n* =32, accession numbers indicated in **S7 Fig**.) were then hierarchically clustered thanks to profile-profile HMM comparisons on their whole proteomes using VirClust (https://rhea.icbm.uni-oldenburg.de/virclust/; version 2, default parameters)."

We included the following paragraph in the result section:

Line 303: "The complete nucleotide sequence of Samy exhibits no substantial similarity to any other deposited phages using BLASTn (with more than 70% nucleotide identity across the genome), indicating its classification as a new phage genus. Accordingly, Samy stands as a singleton in the Actinobacteriophage Database (https://phagesdb.org/phages/Samy/), representing the exclusive member of a distinct cluster identified in a comprehensive analysis of actinobacterial prophages conducted by Sharma *et al.* (26). To delve deeper, we employed specialized tools for clustering phages by comparing their entire proteomes. A proteomic tree created by ViPTree placed Samy within a cluster primarily consisting of *Streptomyces* and *Arthrobacter* phages, albeit on a separate branch (**S7.A Fig.**, similarity scores (S_G) <0.05). The 32 actinophages within this group underwent further analysis using VirClust, resulting in 29, including Samy and PhiC31, being clustered together into a viral genome cluster (VGC, **S7.B Fig.**). Phages within this VGC shared 4 protein superclusters (PSCs), corresponding to the recombination directionality factor (RDF, excisionase), deoxynucleoside monophosphate kinase (DNK), DNA primase and DNA polymerase. The VirClust parameters applied here followed recommendations for delineating tailed phage taxa at the family level (44). However, Samy exhibited a lower number of shared PSCs compared to other phages within the VGC, and its silhouette width value approaching 0 introduces uncertainty regarding its precise taxonomic classification at this time."

As proposed by the referee, we also conducted an analysis using VIRIDIC which confirmed these analyses: The genomic distances calculated by VIRIDIC between Samy and with its closest viruses is significantly below the threshold typically used to define species (95%) or genus (70%). The results are presented in the **Figure R2** which was not included in the revised version since it was redundant with the analysis performed with BLASTn, VIPTree and VirClust.

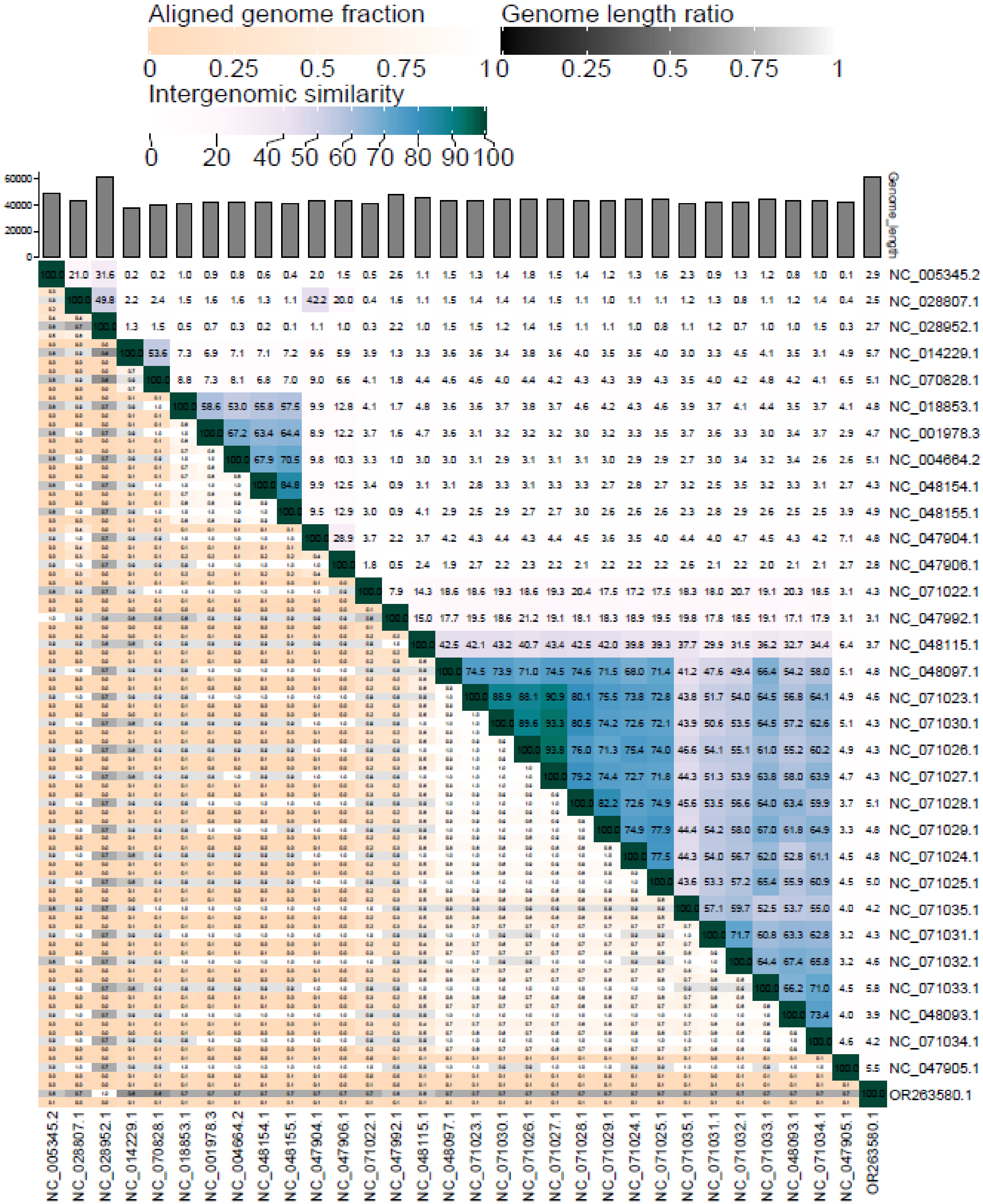

Figure R2: Heatmap incorporating the intergenomic similarity values and alignment indicators of a panel of 32 viruses including Samy

The heatmap was generated by VIRIDIC pipeline applied to the list of 32 phage sequences retrieved from VIPTree analysis (**S7 Fig**.). Samy phage sequence corresponds to 'OR263580.1' (i.e. last line and last column). The phage genome clustering based on intergenomic similarity is indicated in the right half, with darker shades indicating closer relationships. Each number represents the similarity value

for respective genome pairs. On the left half, three indicators are provided for each genome pair: (top) aligned fraction of genome 1, corresponding to the row; (middle) genome length ratio for the two genomes in the pair; (bottom) aligned fraction of genome 2 associated with the column. Darker colors highlight low values, indicating either minimal genome alignment (from orange to white gradient) or substantial differences in genome lengths (from black to white gradient).

- Did the authors test if the CFU's that could be counted after Samy induction still have Samy in there genome? Or are these surviving parts of the hyphae that did not undergo lysis and therefore Samy induction? A bit more speculation on how the authors think that Samy causes these smaller fragments without killing the whole population would benefit the discussion greatly. In line 442 the authors propose that Samy induction in a part of the colony contributes to strain dispersal, but why is Samy only induced in a part of the colony? Please elaborate.

Our answer: Yes, we had actually tested this question but didn't mention the result in the first version of the manuscript. Driven by reviewer's remark, we added the corresponding experiment as an extra figure (**S10 Fig**). Samy prophage is still present in the genomic DNA of strains that survived a 4-day incubation in BM medium. Thus, we propose that the survivors are bacteria that have not undergo Samy induction and therefore did not lyse. The reason for Samy being induced only in a part of the colony remains to be studied. This phenomenon may stem from the heterogeneous distribution of the inducing signal within the multicellular colony and/or from stochastic gene expression.
We have elaborated on this aspect as followed:
Line 369: "The presence of the Samy prophage analysis within the genomes of 'surviving' clones was confirmed by PCR (**S10 Fig**). This suggests that the colonies which persisted did not arise from bacteria that had lost the prophage, but rather from those that had not entered the lytic cycle. "

- In Figure 2C, the difference between plates and liquid should be made more clear. Perhaps put a small line between MM and MP5 to make this distinction more visual.

Our answer: We did the proposed modification in the Figure 2A.

- The microscopy images in Figure 4C and supplementary figure 8A and B are very difficult to see. Is it possible to improve these pictures?

Our answer: We improved the brightness of the images obtained with the Olympus BX63 microscope for the WT strain, whose smaller aggregates were difficult to visualize in the initial images.

Point-by-point reply to referee #2

Referee #2 stated:
The authors report an inducible prophage (Samy) found in the genome of *Streptomyces ambofaciens*. Infectivity of the induced phage virions was confirmed on strains lacking the integrated prophages. The authors performed a comprehensive transcriptome analysis to identify media on which Samy is induced. Finally, they show that prophage induction boosters the dispersal of *Streptomyces*, leading to multicellular aggregates of smaller size. Therefore, the study provides interesting - yet not mechanistic - insights into the impact of prophage induction on the lifestyle of multicellular bacteria.
Our response: We thank the referee for this accurate summary and the positive evaluation of our study.

And then the referee 2 stated:

Comments

General comments:

1) My major concern is that the study lacks any conclusive mechanistic insights or looks further into the broader relevance of this phenomenon (prophage induction in other *Streptomyces* strains).

Our response: Yes, the purpose of this short report is mainly to describe an interesting observation concerning the fact that the prophage induction can have a (somewhat unexpected) effect leading to an increase in the dispersion of strains likely to increase cell survival rather than decrease it. We anticipate that other multicellular bacterial prophages, not necessarily responding to the same signals and/or having distinct replication properties, are likely to be able to have similar effects if their lytic production takes place in only part of the colony (irrespective of their possible specificities). To address this referee's comment and provide some mechanistic evidence for this general concept, we conducted experiments to i) explore the role of NAG in prophage induction, and ii) further describe viral replication within the colony.

2) The impact of prophage induction on multicellular aggregates is interesting, yet not surprising. I believe that induction of Samy is highly heterogeneous and only happens in parts of the mycelium, leading to fragmentation. A visualization of this induction pattern, e.g. by FISH using phage-specific probes, would be highly interesting.

Our response: Driven by this referee's comment, we built a strain encoding the major capsid protein of Samy fused to mCherry (MCP-mCherry). Interestingly, within the colony, we observed a distinct red fluorescence emanating from parts of the filaments, as illustrated in **Fig. R3**. These findings have been incorporated into the revised manuscript (**Fig.4E** and **S9.C Fig.**). This indicates that only a small proportion of the colony produces virus, in addition to other observations already reported (such as a low impact on antibiotic production, the presence of Samy in surviving colonies, etc.).

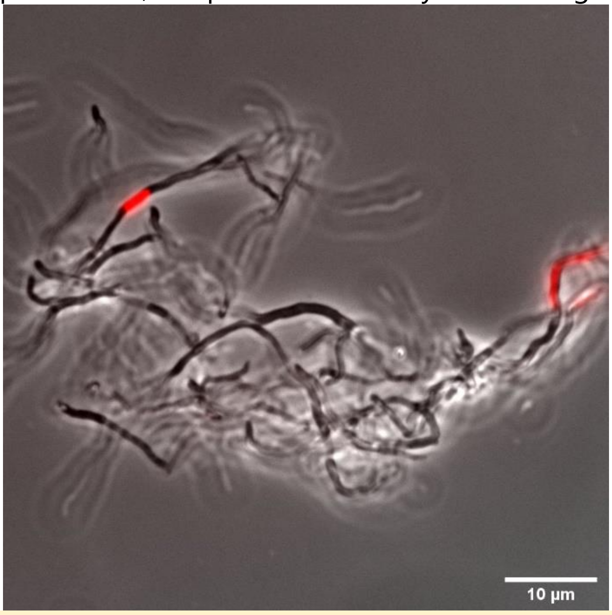

Figure R3: Expression of Samy MCP-mCherry within filaments of *S. ambofaciens* ATCC 23877 bacteria grown 4 days in BM medium

Accordingly, we modified the manuscript as followed:

Results _ Line 372: "To assess the induction pattern, we engineered a strain that expresses the major capsid protein of Samy fused to mCherry (MCP-mCherry). Red fluorescence was observed only in

some segments of the filaments (**Fig. 4.E, S9.C Fig.**), suggesting that Samy phage production was localized to certain areas of the colony and retained within specific filament sectors."

Discussion _ Line 495: "Moreover, Samy production monitored by a MCP-mCherry fusion was detected only in some sections of the colony (**Fig. 4E, S9C Fig.**)."

M&M section: Description of the cloning procedure and microscopy analyses.

3) The authors perform a comprehensive screening of different media conditions leading to prophage induction. However, they do not really nail it down to specific factors leading to induction. They find that N-acetyl-glucosamine is present in the NAG condition and might be the responsible factor. This would be straightforward to check by adding it to non-inducing conditions.

Our response: The impact of NAG added on MM (minimal medium mannitol) solid medium on phage expression is already included in the paper (**Fig. 2**). In accordance with the referee's suggestion, we investigated the influence of NAG on Samy phage production during growth in MM or BM liquid medium. We found that NAG stimulates Samy production in MM liquid medium (**Figure R4**), in accordance with the induction of its expression on MM solid medium (**Fig. 2**). However, NAG has an inhibitory effect on phage production in BM (**Figure R4**). These contrasting outcomes suggest a nuanced role for NAG as a potential signaling molecule for Samy production, albeit with a complex signaling pathway that may vary under different conditions. Due to the lack of a straightforward interpretation of these results regarding the effect of NAG, we opted not to include them in the revised version of the manuscript.

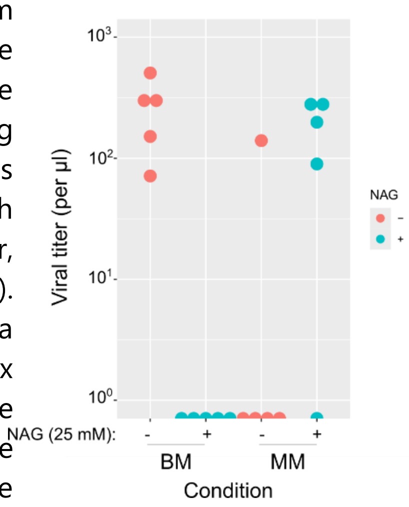

Figure R4: Impact of NAG (25 mM) on phage production by *S. ambofaciens* ATCC 23877 grown in MM or BM medium. Analysis performed by qPCR (as described in the article). Each dot represents an independent experiment.

4) Notably, the authors do not comment on the phylogenetic and taxonomic classification of the newly identified phage. Strikingly, no closest relative could be determined by blast search using the complete genome. Using single proteins, e.g. DNA polymerase/primase, terminase large subunit, major head protein, tail length tape measure protein, endolysin, tail fiber protein or immunity repressor it is interesting to note that these share highest similarities with phages infecting very different species. Best hits include *Streptomyces, Gordonia, Mycobacterium* and *Arthrobacter* phages. Therefore, it would be very interesting to test, if Samy could indeed infect any of these and at least discuss these circumstances.

Our response: This remark about classification is common to both the referees 1 and 2. We conducted an extensive analysis of Samy's classification (see Response to Referee 1), revealing it to be a novel species within a new genus. Furthermore, our infection tests indicated a relatively confined host spectrum, even within the *Streptomyces* genus. Regrettably, lacking strains from the other mentioned genera, we were unable to assess their susceptibility to infection in our laboratory.

Specific comments:
Line 65: A more general, systematic analysis of prophage abundance in *Streptomyces* genomes is provided by Sharma et al., 2023 (https://doi.org/10.1038/s41598-023-30829-z). In contrast to only 7 strains, here a total of 314 Streptomyces genomes were analysed and resulted in 62% containing prophages.
Our response: Yes, we replaced the initial information by a reference to the study of Sharma *et al.*

Line 81: The authors directly jump into *S. ambofaciens*, without explaining why this actually is an interesting model or prophage to study.
Our response: We included a brief section to highlight the significance of studying *S. ambofaciens* within the context of this research.
Sentences added in the introduction:
Line 78: "In this study, we investigated the (pro)phage biology of *Streptomyces ambofaciens*, a strain valued for its spiramycin production. Although the central chromosome region is considered as a hotspot for integrative element insertion (29), this strain is predicted to harbor a complete prophage in its terminal chromosome compartment. We previously reported that in exponential phase, this compartment is less active transcriptionally compared to the central region and exhibits higher levels of antisense-oriented transcription, especially in the predicted prophage region (30). Here, we characterize the condition of production of this novel temperate phage from *Streptomyces ambofaciens* ATCC 23877, which we named 'Samy'."

Fig 2B: I think it is difficult to compare gene expression between solid and liquid growth plus using two different time points for comparison. In this experiment, strongest induction was observed on solid media. Is induction on solid media in general more pronounced? But there no direct comparison on liquid and solid media for any of the media/conditions, which I found surprising.
Our response: We agree with the Referee 2 that the transcriptome work plan is not completely equilibrated. Indeed, it was initially established as part of an OSMAC ("one strain many compounds") approach aimed at characterizing a new antibiotic activity (data not published). During these experiments, we happened to observe phage induction, prompting us to delve into its impact on *Streptomyces* physiology.

Line 286 ff: Can Samy also lysogenize *S. lividans*? Or if the infection of this strain is strictly lytic.
Our response: In this study, we did not investigate the phage cycle in other strains. However, in a parallel project, we cloned the Samy phage integrase to generate an integrative tool. We observed that this vector could integrate efficiently in *S. lividans*, suggesting that the phage might be capable of initiating a lysogenic lycle in this strain. This work will be the subject of a separate publication.

Point-by-point reply to referee #3

Referee #3 stated:
Jaffal and colleagues identified a prophage in *Streptomyces ambofaciens* that upon induction leads to dispersal of bacteria. The phage, named "Samy", was identified through differences in GC content (66%) and annotated using PHASTER. Through different growth conditions (e.g. different media, alkaline growth conditions, different growth phases), the authors concluded that Samy is expressed during a general stress response, although this seems to be independent of the SOS response (with the signals for induction yet to be determined). Growth data indicate a complex role of Samy in

influencing *Streptomyces* growth dynamics, particularly under stress conditions, by preventing large cell aggregate formation and promoting bacterial dispersal, without adversely affecting antibiotic production. All of this indicates that phage production is part of a more general stress and dispersal response of *S. ambofaciens* but the role of this phage in growth, division, adaptation to environmental niches, production of antibiotics and other compounds, has not been studied.
In summary, the authors present interesting observations and describe a novel prophage together with its genetic and metabolic pathways involved. However, the precise mechanisms and signals leading to the production and release of phages, and the biologic role this mechanism plays during the bacterial life cycle are not explained and the study therefore remains descriptive.

Our response: We agree with Referee 3 regarding the lack in our study of detailed explanation regarding the mechanisms driving phage production and release. We explored the impact of phage induction on antibiotic production, and found minimal influence. Our main focus is to pioneer the description of the influence of prophage production on *Streptomyces* colony dispersal. This marks an original observation, particularly regarding the role of phages under stress conditions. In order to enrich the manuscript regarding mechanistic aspects, we explored the question of the impact of NAG on phage induction (mitigate effect – please reference to **Figure R4**), and of the proportion of cells expressing the phage major capsid protein. These latter experiments provide a better description of the phage cycle in a *Streptomyces* colony under stress, and constitute a first step towards more mechanistic studies.
